# Therapeutic Potential of Cucurbitacin I in Colon Adenocarcinoma Is Mediated by Modulation of SDHA Expression

**DOI:** 10.1111/jcmm.71231

**Published:** 2026-06-09

**Authors:** Danni Zhao, Xunzhi Zhang, Wenke Xiao, Yue Lin, Zhixiang Wu, Xinyu Tang, Qian Cheng, Pengmian Feng, Wei Chen

**Affiliations:** ^1^ School of Intelligent Medicine Chengdu University of Traditional Chinese Medicine Chengdu China; ^2^ School of Basic Medicine Chengdu University of Traditional Chinese Medicine Chengdu China; ^3^ School of Pharmacy Chengdu University of Traditional Chinese Medicine Chengdu China; ^4^ Innovative Institute of Chinese Medicine and Pharmacy Chengdu University of Traditional Chinese Medicine Chengdu China; ^5^ Institute of Herbgenomics Chengdu University of Traditional Chinese Medicine Chengdu China

**Keywords:** colon adenocarcinoma, cucurbitacin I, landscape dynamic network biomarker methodology, progression biomarker, succinate dehydrogenase complex flavoprotein subunit A

## Abstract

Colorectal cancer is a prevalent malignancy, with colon adenocarcinoma (COAD) representing its most common histological subtype. Although most stage I patients remain disease‐free after treatment, a subset rapidly progresses to advanced disease with markedly reduced survival. Therefore, identifying early molecular warning signals in stage I patients is essential for timely intervention. In this study, integrated datasets from The Cancer Genome Atlas and Genotype‐Tissue Expression projects were analysed using the landscape dynamic network biomarker (*l*‐DNB) approach to identify progression biomarkers (PBs) for COAD. Twenty PBs were identified, among which succinate dehydrogenase complex flavoprotein subunit A (SDHA) was selected for further investigation. The L1000CDS^2^ database was subsequently queried utilizing these identified PBs, identifying Cucurbitacin I (CuI) as a promising therapeutic component against COAD. Molecular docking and molecular dynamics simulations demonstrated stable binding between CuI and SDHA, with a binding energy of −9.44 kcal/mol and an RMSD of 2.0 ± 0.3 Å. Subsequent in vitro experiments demonstrated that CuI treatment upregulated SDHA expression and inhibited activation of the NF‐κB pathway. Collectively, these findings suggest that the identified PBs may serve as early‐warning indicators for stage I COAD patients and that CuI suppresses COAD cell proliferation, potentially through the SDHA/NF‐κB axis, highlighting its promise as a potential therapeutic candidate.

**Abbreviations**
CI: Confidence interval
COAD: Colon adenocarcinoma
CRC: Colorectal cancer
CRLS1: Cardiolipin synthase 1
CuI: Cucurbitacin I
DEGs: Differentially expressed genes
FC: Fold change
GO: Gene Ontology
GTEx: Genotype‐Tissue Expression
HPA: Human Protein Atlas
HR: Hazard ratio
$I_{\mathrm{DNB}}$
: Global dynamic network biomarker score
$I_{s}$
: Local dynamic network biomarker score
KEGG: Kyoto Encyclopedia of Genes and Genomes
L1000CDS^2^
: LINCS L1000 characteristic direction signatures search engine
*l*‐DNB: Landscape dynamic network biomarker
LZTR1: Leucine zipper transcription regulator‐like 1
MD: Molecular dynamics
NDUFA9: NADH ubiquinone oxidoreductase subunit A9
PBs: Progression biomarkers
PCA: Principal‐component analysis
PSMA6: Proteasome subunit α 6
Rg: Radius of gyration
RMSD: Root mean square deviation
RMSF: Root mean square fluctuation
ROS: Reactive oxygen species
SDHA: Succinate dehydrogenase complex flavoprotein subunit A
TCGA: The cancer genome atlas
TCM: Traditional Chinese medicine

## 1 Introduction

Colorectal cancer (CRC) is the third most common cancer and the second leading cause of cancer‐related mortality worldwide [1, 2]. Colon adenocarcinoma (COAD), the predominant histological subtype, accounts for more than 90% of CRC cases [3, 4]. Despite advances in screening, approximately 20% of patients present with distant metastases at initial diagnosis, and their five‐year survival rate remains below 15% [5, 6]. In contrast, patients with stage I COAD achieve postoperative five‐year survival rates exceeding 90% [7], underscoring the critical importance of early detection and timely intervention as the most cost‐effective strategies for reducing disease burden. However, early‐stage diagnosis does not uniformly translate into favourable outcomes. Even among stage I patients, substantial heterogeneity exists in progression risk. While most individuals remain disease‐free, a subset rapidly progresses to advanced stages associated with sharply reduced survival. This clinical paradox underscores a critical unmet need to identify molecular warning signals in stage I that can predict imminent progression before irreversible deterioration occurs.

To achieve this goal, it is essential to move beyond the constraints of static diagnostic markers and instead prioritize dynamic molecular indicators that can capture early transitional states preceding clinical progression. The Dynamic Network Biomarker (DNB) framework, grounded in non‐linear dynamical systems theory, has been developed to detect critical transition states in disease progression [8]. The landscape dynamic network biomarker (*l*‐DNB) approach constitutes a refined and model‐free extension, facilitating the detection of pre‐transition signals utilizing single‐sample omics data [9]. By quantifying network perturbations at the individual level, *l*‐DNB detects genes exhibiting maximal local criticality scores, which serve as candidate biomarkers for impending phenotypic progression. Unlike conventional biomarkers that distinguish normal from disease states, *l*‐DNB captures unstable tipping‐point states in which quantitative molecular alterations precipitate qualitative disease transitions, thereby revealing potential windows for reversible intervention. Previous studies have demonstrated its applicability across multiple cancer contexts [10, 11, 12], supporting its potential for identifying progression‐associated signals in early‐stage COAD.

Beyond risk stratification, progression biomarkers (PBs) identified through *l*‐DNB analysis may also uncover functionally critical molecular drivers of early disease progression. Given that metabolic reprogramming is a hallmark of cancer, dysregulation of metabolic pathways represents a plausible mechanism contributing to early tumour evolution. In this context, succinate dehydrogenase complex flavoprotein subunit A (SDHA) stands out due to its dual role in energy metabolism and its established link to tumour suppression in other cancers [13, 14, 15]. As a key component of both the mitochondrial respiratory chain and the citric acid cycle, SDHA catalyses a critical reaction linking the tricarboxylic acid cycle to the electron transport chain, thereby maintaining cellular bioenergetic homeostasis [16]. While normal cells rely on SDHA to maintain metabolic equilibrium, cancer cells frequently reprogram metabolic pathways, potentially disrupting SDHA activity and promoting tumour progression and metastasis [17, 18, 19]. Although SDHA mutations and dysregulation have been extensively characterized in pheochromocytomas, paragangliomas and gastrointestinal stromal tumours [20], its functional role in COAD remains elusive.

Exploiting SDHA‐mediated metabolic vulnerabilities requires therapeutic components capable of modulating mitochondrial bioenergetics. Natural components derived from traditional Chinese medicine (TCM) represent a rich source of candidate anticancer components [21, 22]. Cucurbitacin I (CuI), a natural tetracyclic triterpenoid isolated from Cucurbitaceae plants, has attracted attention for its anticancer properties [23, 24]. Traditionally used for antipyretic, analgesic, anti‐inflammatory and antimicrobial purposes, CuI has been shown in vitro to inhibit proliferation, migration and invasion while inducing cell‐cycle arrest and apoptosis [25]. In addition, CuI has been shown to trigger cytoprotective autophagy in glioblastoma cells [26], and suppress progression of non‐small‐cell lung cancer cells [27]. These findings suggest that CuI may represent a promising candidate for further investigation in the context of stage I COAD.

**FIGURE 1:**
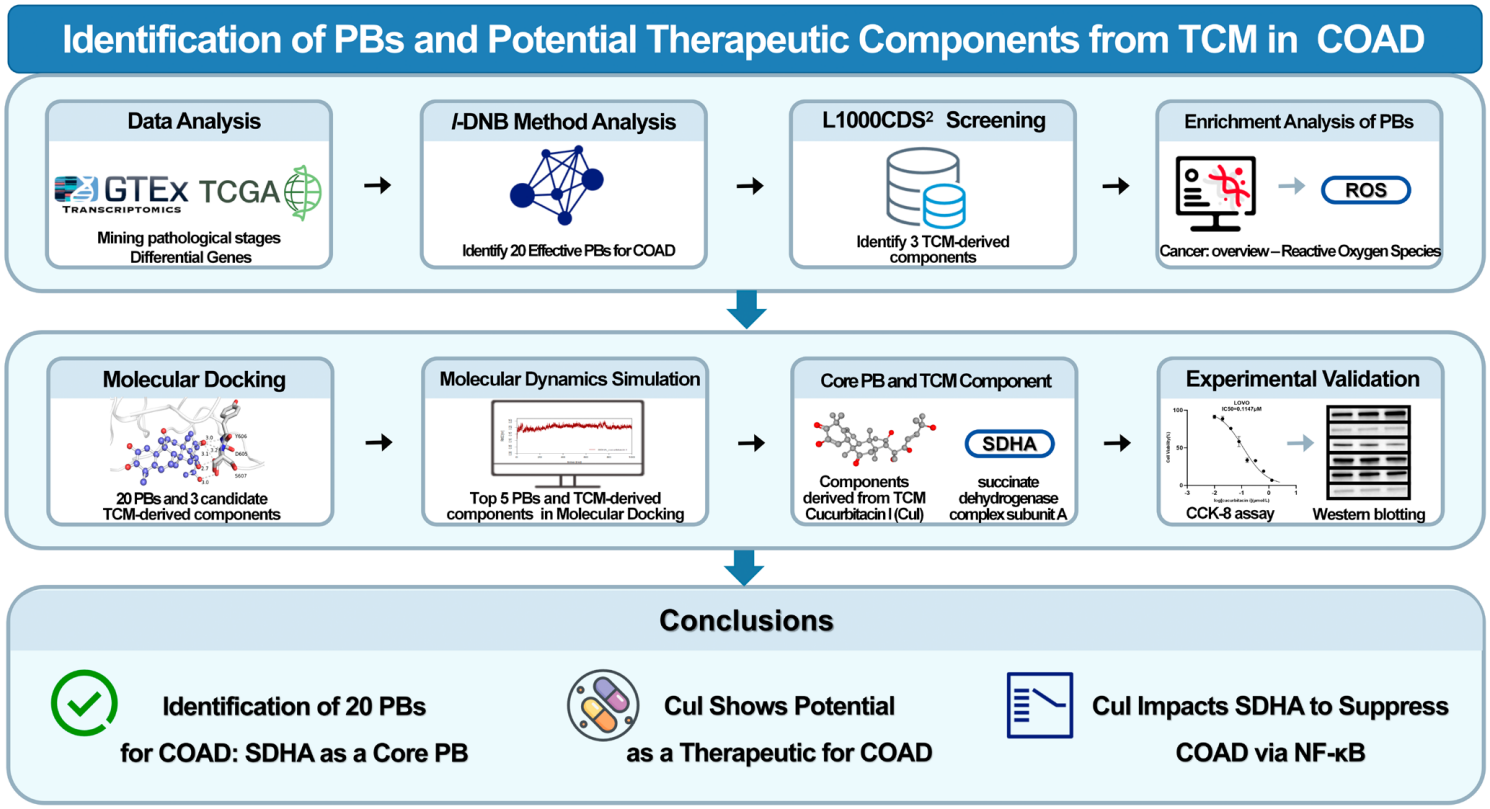
Schematic diagram of architecture of the study. The architecture consists of three parts. Screening of PBs and prediction of TCM‐derived components: Based on GTEx and TCGA data, the *l*‐DNB method was employed to identify 20 PBs for COAD. Three components derived from TCM were screened using the L1000CDS^2^. Computational simulation validation: Molecular docking and MD simulations were performed to investigate the interactions between PBs and the components, confirming the core targeting relationship between SDHA and CuI. Experimental validation: In vitro experiments were conducted to validate that CuI inhibits COAD progression by upregulating SDHA expression and suppressing NF‐κB activation. COAD, colon adenocarcinoma; CuI, cucurbitacin I; GTEx, Genotype‐Tissue Expression; L1000CDS^2^, LINCS L1000 characteristic direction signatures search engine; *l*‐DNB, landscape dynamic network biomarker; MD, molecular dynamics; PBs, progression biomarkers; SDHA, succinate dehydrogenase complex flavoprotein subunit A; TCGA, The Cancer Genome Atlas; TCM, traditional Chinese medicine.

Building on this rationale, the present study establishes an integrated framework linking PB‐guided discovery with therapeutic evaluation (Figure 1). By combining dynamic biomarker identification, characterization of SDHA as a key metabolic node and systematic exploration of CuI, we aim to elucidate the molecular mechanisms underlying stage I COAD progression and to identify potential points of intervention. This multi‐level strategy provides a coherent platform for translating insights from PBs into candidate therapeutic opportunities for early‐stage COAD.

## 2 Materials and Methods

### 2.1 Gene Expression Analysis

Gene expression data for COAD and normal colon tissues were obtained from The Cancer Genome Atlas (TCGA) [28] and the Genotype‐Tissue Expression (GTEx) [29]. After quality filtering, 308 normal colon samples from GTEx were included. To mitigate batch effects across different datasets, between‐array normalization was applied using the normalizeBetweenArrays function from the limma R package. Principal component analysis (PCA) was conducted both before and after normalization to evaluate the effectiveness of batch correction in reducing inter‐dataset variation. Differential expression analysis was performed using the R packages limma (v 3.58.1) and edgeR (v 4.0.16). Genes with |log_2_ FC| > 2.5 and *p* < 0.01 were considered differentially expressed genes (DEGs). Volcano plots and heat maps of DEGs were created using the ggplot2 (v 3.5.1) and pheatmap (v 1.0.12) packages.

### 2.2 Identification of PBs and Critical State Using the *l*‐DNB Method

The *l*‐DNB method was utilized to identify PBs and determine the critical state of COAD progression [9]. According to the original *l*‐DNB framework, a reference network was first constructed using Pearson Correlation Coefficients (PCC) computed across all normal colon tissue samples from the GTEx database. For each COAD sample from the TCGA‐COAD dataset, SSN was generated by quantifying the perturbation introduced by that individual sample to the reference co‐expression network. Specifically, the single‐sample Pearson Correlation Coefficient (sPCC) was defined as the difference between the Pearson correlation recalculated after adding the tumour sample to the reference set and the original reference correlation. Gene pairs with statistically significant sPCC values [9, 30] were retained as edges in the SSN. For each sample, genes were then ranked according to their local DNB score ($I_{s}$), and the top 20 genes were selected as candidate PBs. Subsequently, the mean $I_{s}$ of these 20 genes was calculated to obtain the global DNB score ($I_{\mathrm{DNB}}$) for each sample. Samples were then stratified by pathological stage, and the stage with the highest average $I_{\mathrm{DNB}}$ was identified as the critical state, reflecting maximal network instability and corresponding to a tipping point preceding qualitative phenotypic progression [30]. In COAD, stage I exhibited the highest $I_{\mathrm{DNB}}$, suggesting that this stage represents a critical window wherein molecular heterogeneity may determine progression risk. The top 20 genes ranked by the highest $I_{s}$ from samples within this critical stage were designated as the final identified PBs. A detailed procedure is provided in the Methods S1.

### 2.3 Functional Enrichment Analysis of PBs

Gene Ontology (GO) and Kyoto Encyclopedia of Genes and Genomes (KEGG) analyses were performed to identify overrepresented biological processes, molecular functions and signalling pathways. These analyses were implemented by using clusterProfiler (v 4.10.1), org.Hs.eg.db (v 3.18.0), enrichplot (v 1.22.0) and GOplot (v 1.0.2) packages. The results were visualized as chord diagrams using the circlize (v 0.4.16) and ggplot2 (v 3.5.1) packages.

### 2.4 Screening of TCM‐Derived Components

The dysregulated PBs were subsequently used to query against the LINCS L1000 characteristic direction signatures search engine (L1000CDS^2^) [31] to screen for natural components capable of reversing disease‐associated transcriptomic signatures. Components were ranked according to the percentage of reversed genes, and the top 50 candidates were highlighted, enabling rational prioritization of TCM‐derived components for further evaluation.

### 2.5 Molecular Docking of TCM‐Derived Components With PBs

Mol2 files for the ligands (CuI, Rottlerin, RHODOMYRTOXIN) were sourced from the PubChem database [32]. The 3D structures of the target proteins (20 PBs) were downloaded in PDB format from the RCSB PDB database [33]. Proteins were prepared using PyMOL (v 3.0.4) by removing water molecules and native ligands, and by adding hydrogen atoms. The ligand was converted to docking‐compatible formats using OpenBabel (v 2.4.0). Molecular docking was conducted with AutoDock (v 1.5.7) to calculate binding energies. Protein‐ligand interaction patterns were visualized in PyMOL (v 3.0.4).

### 2.6 Molecular Dynamics Simulation

To evaluate the temporal stability of high‐affinity protein‐ligand complexes, molecular dynamics (MD) simulations were performed using GROMACS 2021.7 [34] with the CHARMM36 all‐atom force field [35] and CHARMM General Force Fields (CGenFF) [36] for protein and ligand parameters, respectively. Ligand information for CuI was generated using the CGenFF server (https://cgenff.com). Five top‐scoring complexes (SDHA‐CuI, LZTR1‐CuI, CRLS1‐CuI, PSMA6‐CuI and NDUFA9‐CuI) were solvated in a cubic water box with periodic boundary conditions and neutralized with Na^+^/Cl^−^ ions. Energy minimization was performed for 5000 steps using the steepest‐descent algorithm with a step size of 0.01 nm. The temperature was increased to and maintained at 300 K through a 100 ps NVT simulation employing the V‐rescale thermostat, followed by a 100 ps NPT simulation with the Parrinello‐Rahman barostat to stabilize the pressure at 1 bar. Finally, a 100 ns production run was performed, saving trajectories every 10 ps for analyses such as root mean square deviation (RMSD), root mean square fluctuation (RMSF) and radius of gyration (Rg).

### 2.7 Survival and Expression Analysis of SDHA in COAD

SDHA expression levels were extracted from integrated TCGA‐COAD and GTEx normal colon tissue datasets, and patients were divided into high‐ and low‐expression groups based on the median expression value. Kaplan–Meier survival curves were generated using the R survival (v 3.8.1) package, and visualization was achieved with the survminer (v 0.5.0) package, which also calculated hazard ratios (HR) and 95% confidence intervals (CI). Statistical significance was evaluated through log‐rank tests. Differences in SDHA expression between tumour and normal tissues were analysed using two‐tailed Student's *t*‐tests, and expression patterns were visualized with boxplots.

### 2.8 Cell Lines and Cell Cultures

The LOVO, HCT116 and HT29 cell lines used in this study were obtained from the Cell Bank of the Chinese Academy of Sciences (CBCAS, Beijing, China) and cultivated in Dulbecco's Modified Eagle Medium (DMEM: Gibco, BOS, USA) supplemented with 1% penicillin/streptomycin (PS: Gibco, BOS, USA) and 10% fetal bovine serum (FBS: Vicacell, Shanghai, China). Cells were incubated in T25 flasks (Thermo Fisher, BOS, USA) at 37°C with 5% CO_2_. Upon reaching ~80% confluence, cells were washed with phosphate‐buffered saline (PBS: Solarbio, Shanghai, China), followed by passage or plating and digested with 0.25% trypsin (Gibco, BOS, USA). Cucurbitacin I (CuI, MCE, NJ, USA) was dissolved in dimethyl sulfoxide (DMSO: Solarbio, Shanghai, China) to prepare stock solutions, and treatment concentrations were adjusted to ensure a final DMSO concentration remained ≤ 0.1% control wells received an equivalent volume of DMSO without CuI. The anti‐proliferative effects of CuI were assessed across a range of concentrations in the three COAD cell lines.

### 2.9 Reagents and Antibodies

SDHA Polyclonal antibody (1:10000; 14865‐1‐AP) and GAPDH Polyclonal antibody (1:5000; 10494‐1‐AP) were obtained from Proteintech Group Inc. (Wuhan, China). NF‐κB p65 (PTR2315) Mouse mAb (1:1000; YM3111) and NF‐κB p65 (Phospho Ser536) Rabbit pAb (1:1000; YP0191) were supplied by ImmunoWay Biotechnology Company (ImmunoWay, TX, USA). The secondary antibody, Horseradish Peroxidase (HRP)‐conjugated Goat Anti‐Rabbit IgG (H + L) (1:5000; RS0002) was supplied by ImmunoWay Biotechnology Company (ImmunoWay, TX, USA). Horseradish Peroxidase (HRP)‐conjugated Goat Anti‐Mouse IgG (H + L) (1:5000; SA00001‐1) was obtained from Proteintech Group Inc. (Wuhan, China).

### 2.10 Western Blotting Assay

RIPA buffer (Beyotime, Shanghai, China) supplemented with phosphatase and protease inhibitors (Selleck, HOU, USA) was used for cell lysis at 4°C for 30 min. The lysates were then centrifuged to collect the supernatants. After electrophoresis, the proteins from the lysates were transferred onto 0.45 μM polyvinylidene fluoride (PVDF) membranes (Millipore, BOS, USA) for blocking, followed by incubation with primary and HRP‐conjugated secondary antibodies. Target proteins were visualized with the Immobilon Western chemiluminescent HRP substrate (Millipore, USA). The Chemiluminescent Imaging System (Tanon, Shanghai, China) was used to capture and analyse the protein blot images.

### 2.11 Cell Viability Assay

Cellular proliferation was evaluated using the Cell viability assay (CCK‐8). Cells were seeded at 8 × 10^3^ per well in a 96‐well plate and exposed to varying CuI concentrations. After 48 h of culture, 100 μL of CCK‐8 reagent was added to each well, and the cells were incubated for an additional 2 h. Optical density was then measured at 450 nm using a multifunctional microplate reader (SpectraMax iD3, USA).

### 2.12 Statistical Analysis

Statistical comparisons were conducted using one‐way ANOVA (cell viability) and nonlinear regression (IC_50_). ImageJ (version 1.54 g) was used for western blot quantification, and statistical analysis was performed with R software (version 4.3.3) using the multcomp package (version 1.4.29) to conduct one‐way ANOVA followed by Dunnett's test. Significance thresholds were defined as *p < 0.05, **p < 0.01, ***p < 0.001, ****p < 0.0001.

## 3 Results

### 3.1 Identification of Stage‐Specific Transcriptomic Signatures in COAD

**FIGURE 2:**
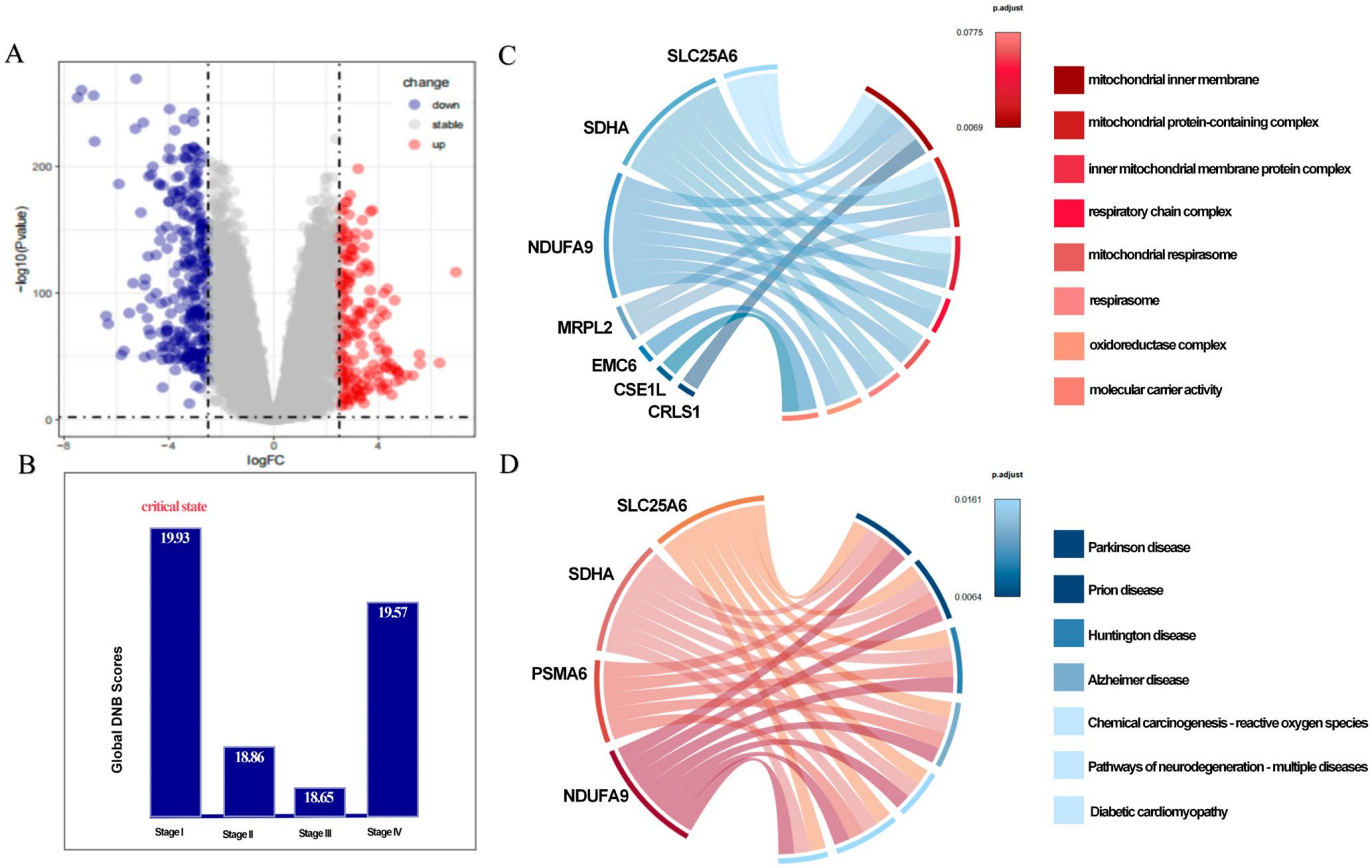
Identification of PBs and their functional annotation. (A) Volcano plot illustrating DEGs in stage I COAD. (B) $I_{\mathrm{DNB}}$ scores across stages I‐IV. Stage I exhibited the highest $I_{\mathrm{DNB}}$ score, indicating maximal molecular network perturbation at this earliest pathological stage and identifying it as a critical state preceding potential progression to advanced disease. (C) GO enrichment analysis of PBs. (D) KEGG pathway analysis of PBs. Colour depth is positively correlated with the statistical significance of enrichment. Abbreviations: DEGs, differentially expressed genes; $I_{\mathrm{DNB}}$, global dynamic network biomarker score; GO, Gene Ontology; KEGG, Kyoto Encyclopedia of Genes and Genomes.

To characterize the molecular landscape of COAD progression, we integrated GTEx normal colon tissues (*n* = 308) with TCGA COAD samples spanning pathological stages I‐IV (*n* = 468 total). Batch correction effectively removed platform‐specific biases (Figure S1A), enabling cross‐cohort comparison. Differential expression analysis revealed stage‐specific signatures, including 445 DEGs in stage I, 437 in stage II, 414 in stage III and 415 in stage IV (Table S1, Figure 2A, Figure S1B,C). Notably, DEG counts remained relatively stable across stages, with a core set of 378 DEGs common to all four stages, the largest overlapping region in the diagram (Figure S2). This indicates that major transcriptomic divergence from normal tissue occurs early and is maintained throughout progression. This observation supports our hypothesis that critical network instability, rather than cumulative mutation burden, distinguishes early‐stage progression risk.

### 3.2 Stage I Represents the Critical Transition State

Implementation of the *l*‐DNB framework uncovered a non‐monotonic trajectory of global network instability throughout COAD progression (Figure 2B). The $I_{\mathrm{DNB}}$ peaked at stage I (19.93), and decreased at stage II (18.86) and stage III (18.65), followed by a modest increase at stage IV (19.57). This pattern is consistent with critical transition theory, in which molecular networks exhibit maximal fluctuation at tipping points preceding qualitative state transitions. The decline of $I_{\mathrm{DNB}}$ from stage I to stages II‐III suggests that once malignant progression is initiated, the system stabilizes in a new state with reduced network plasticity. The modest rebound at stage IV may reflect metastasis‐associated alterations rather than a reversion to the critical transition state. Based on these findings, the top 20 genes ranked by $I_{s}$ at stage I were selected as PBs (Table S2). The choice of this threshold (*k* = 20) was supported by cumulative score and marginal gain analyses (Figure S3), which confirmed that the top 20 genes captured the dominant critical module while minimizing the inclusion of low‐signal noise. The selected PBs exhibited coordinated high criticality ($I_{s}$ range: 13.91–36.11), with PERP, SLC25A6 and CRLS1 ranking highest.

### 3.3 Enrichment of PBs in Mitochondrial Dysfunction and Cancer‐Related Pathways

Functional enrichment analysis of the 20 PBs indicated that mitochondrial bioenergetic disruption represents a hallmark of the stage I critical transition. GO analysis revealed that the enriched terms were predominantly associated with mitochondrial structure and function, including mitochondrial inner membrane, mitochondrial protein‐containing complex, inner mitochondrial membrane protein complex, respiratory chain complex, and molecular carrier activity (Figure 2C). Collectively, the perturbation of these critical nodes in mitochondrial bioenergetics is expected to trigger the accumulation of reactive oxygen species (ROS). This mechanistic expectation was corroborated by KEGG analysis: while multiple entries were related to neurodegenerative diseases, Chemical carcinogenesis‐ROS, which is closely associated with oxidative stress, was significantly enriched among cancer‐related pathways (Figure 2D).

This enrichment pattern is mechanistically coherent. Five PBs (SDHA, NDUFA9, CRLS1, MRPL2, SLC25A6) encode proteins directly localized to mitochondria, participating in oxidative phosphorylation, TCA cycle and mitochondrial quality control [37, 38, 39, 40, 41]. The remaining three PBs (PSMA6, EMC6, CSE1L) are indirectly linked to mitochondrial function through proteostasis [42], ER‐mitochondria crosstalk [43] and energy sensing pathways [44], respectively. The prominence of these PBs further supports the notion that mitochondrial dysfunction generates oxidative stress, creating a feed‐forward loop that destabilizes cellular homeostasis at the critical transition window.

### 3.4 Network Pharmacology Identifies CuI as a Candidate PB‐Targeting Component

**FIGURE 3:**
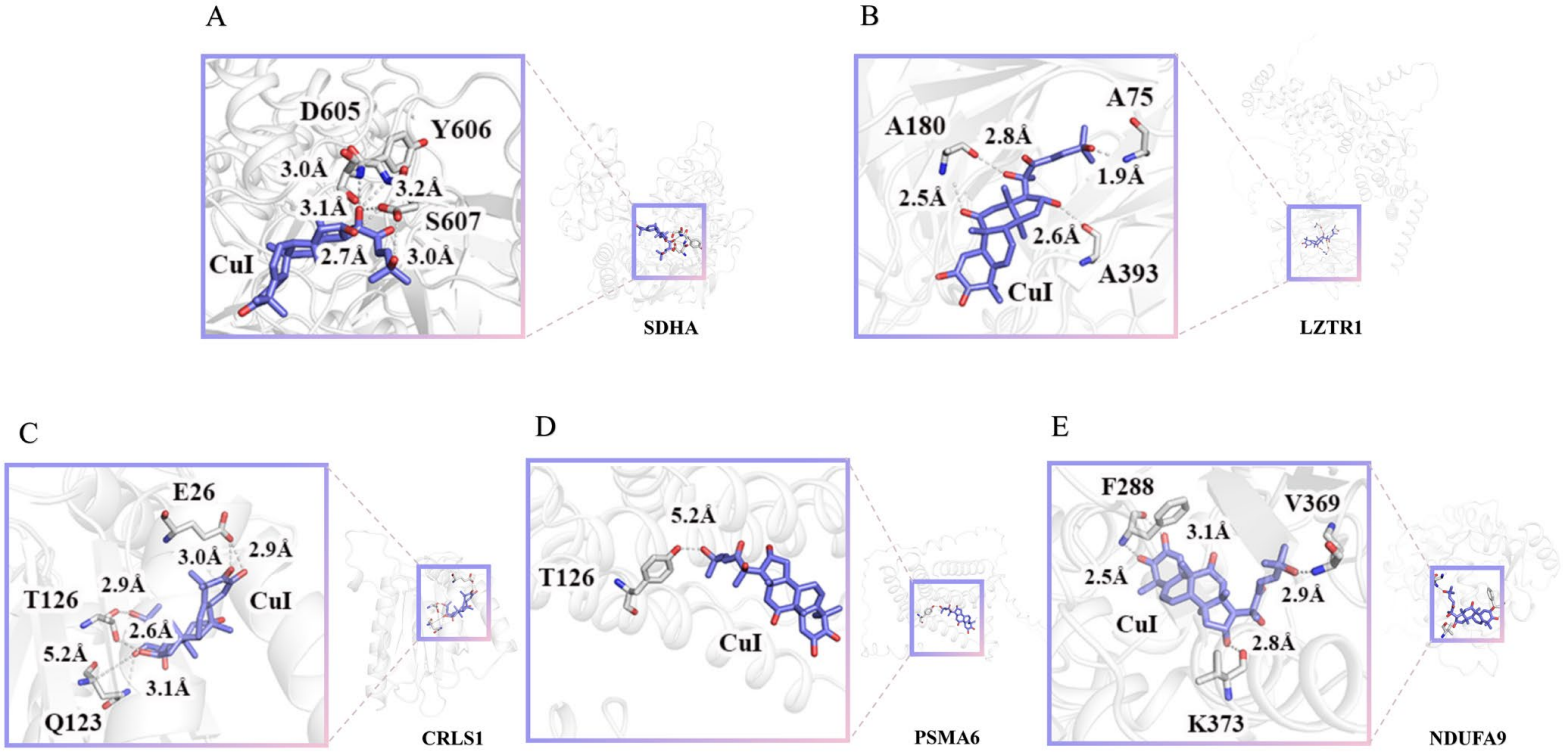
Molecular docking conformations of CuI with the top five PBs. The top five molecular docking results for CuI are illustrated using a stick model. Target proteins are represented as stick models, and key hydrogen bonds are indicated by dashed white lines. Panels (A–E) depict the docking outcomes for CuI with SDHA, LZTR1, CRLS1, PSMA6 and NDUFA9, respectively. CRLS1, cardiolipin synthase 1; LZTR1, leucine zipper transcription regulator‐like 1; NDUFA9, NADH Ubiquinone oxidoreductase subunit A9; PSMA6, proteasome subunit α 6.

To identify components capable of reversing the stage I critical state, we generated a PB‐derived gene signature and performed reverse connectivity mapping using L1000CDS^2^. Among the top 50 hits (Table S3), three TCM‐derived components, namely Rottlerin, CuI and RHODOMYRTOXIN B, were prioritized. Molecular docking across all 20 PBs showed that CuI displayed broad yet structured binding interactions (Table S4). While CuI bound multiple PBs (Table S5), the top five complexes by binding energy all involved CuI, including SDHA (−9.44 kcal/mol), LZTR1 (−8.80 kcal/mol), CRLS1 (−7.92 kcal/mol), PSMA6 (−7.65 kcal/mol) and NDUFA9 (−7.53 kcal/mol) (Figure 3). SDHA, NDUFA9 and CRLS1 are all components of mitochondrial ETC/TCA pathways, suggesting that CuI may coordinately modulate bioenergetic networks rather than acting through single‐target inhibition. CuI was therefore selected for experimental validation based on its strong predicted affinity for SDHA, previously reported antitumor activity [45], and known effects on mitochondrial function [46].

### 3.5 MD Simulation Confirms Stable SDHA‐CuI Interaction

**FIGURE 4:**
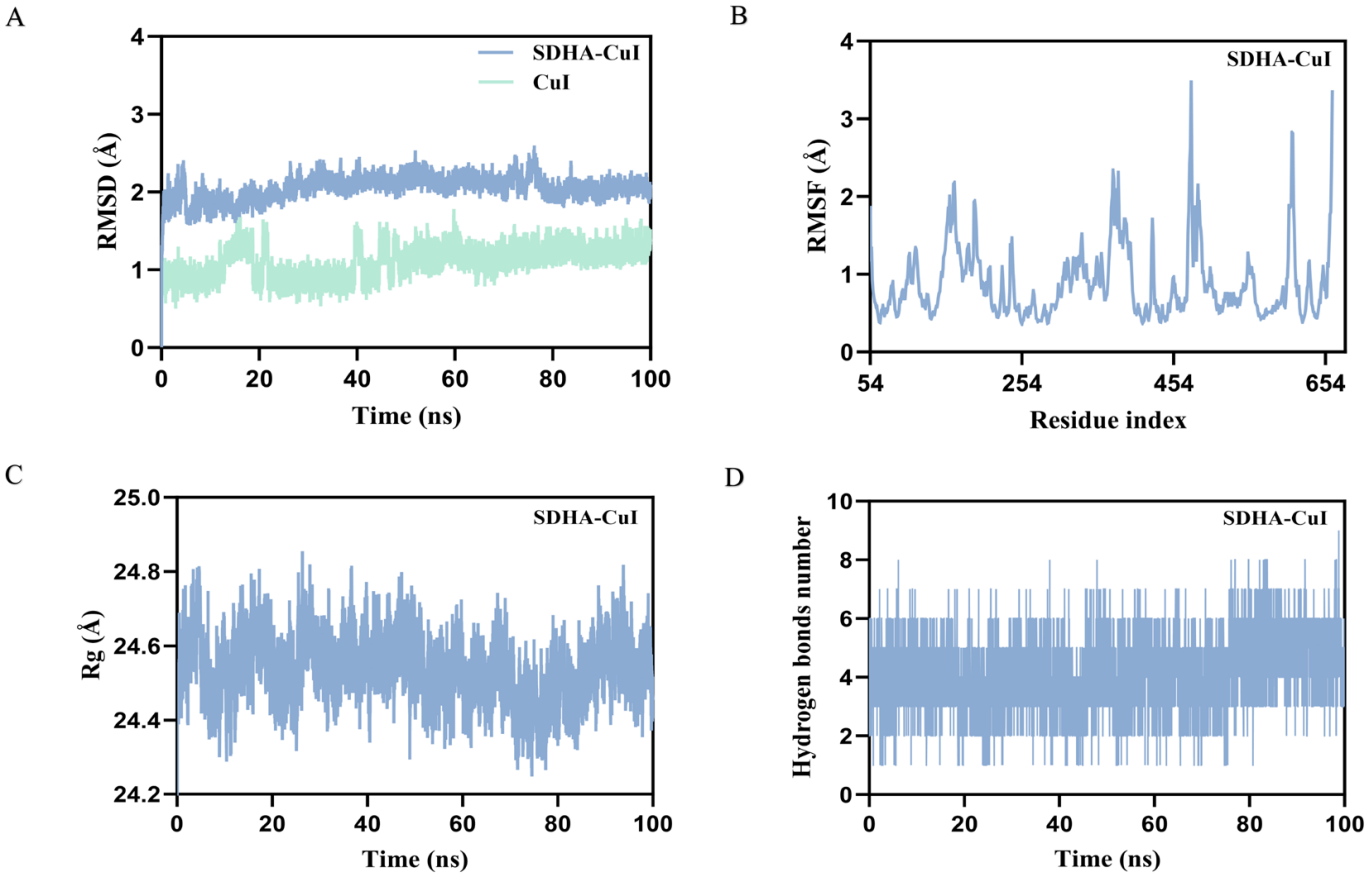
Conformational stability and dynamic behaviour analysis of the SDHA‐CuI complex. (A) RMSD of the SDHA‐CuI complex (blue) and CuI (green) over 100 ns MD simulation. (B) RMSF of protein residues in the SDHA‐CuI complex. (C) Rg of the SDHA‐CuI complex. (D) Number of hydrogen bonds in the SDHA‐CuI complex over the 100 ns MD simulation. MD, molecular dynamics; RMSD, root mean square deviation; RMSF, root mean square fluctuation; Rg, radius of gyration.

MD simulations further supported the stability of the SDHA‐CuI complex (Figure 4). Over a 100 ns trajectory, the complex maintained stable backbone RMSD (2.0 ± 0.3 Å), low RMSF (< 6 Å) and consistent compactness (Rg 24.2 ~ 24.8 Å). An average of 4.0 ± 0.5 hydrogen bonds was formed between CuI and catalytic residues Asp605, Tyr606 and Ser607. Comparison across the top five docking complexes indicated that SDHA‐CuI displayed the highest dynamic stability. Although LZTR1‐CuI and CRLS1‐CuI exhibited similar initial docking energies, they showed greater conformational fluctuation and reduced hydrogen‐bond persistence (Figure S4).

### 3.6 Integrative Validation Identifies SDHA as a Core PB

**FIGURE 5:**
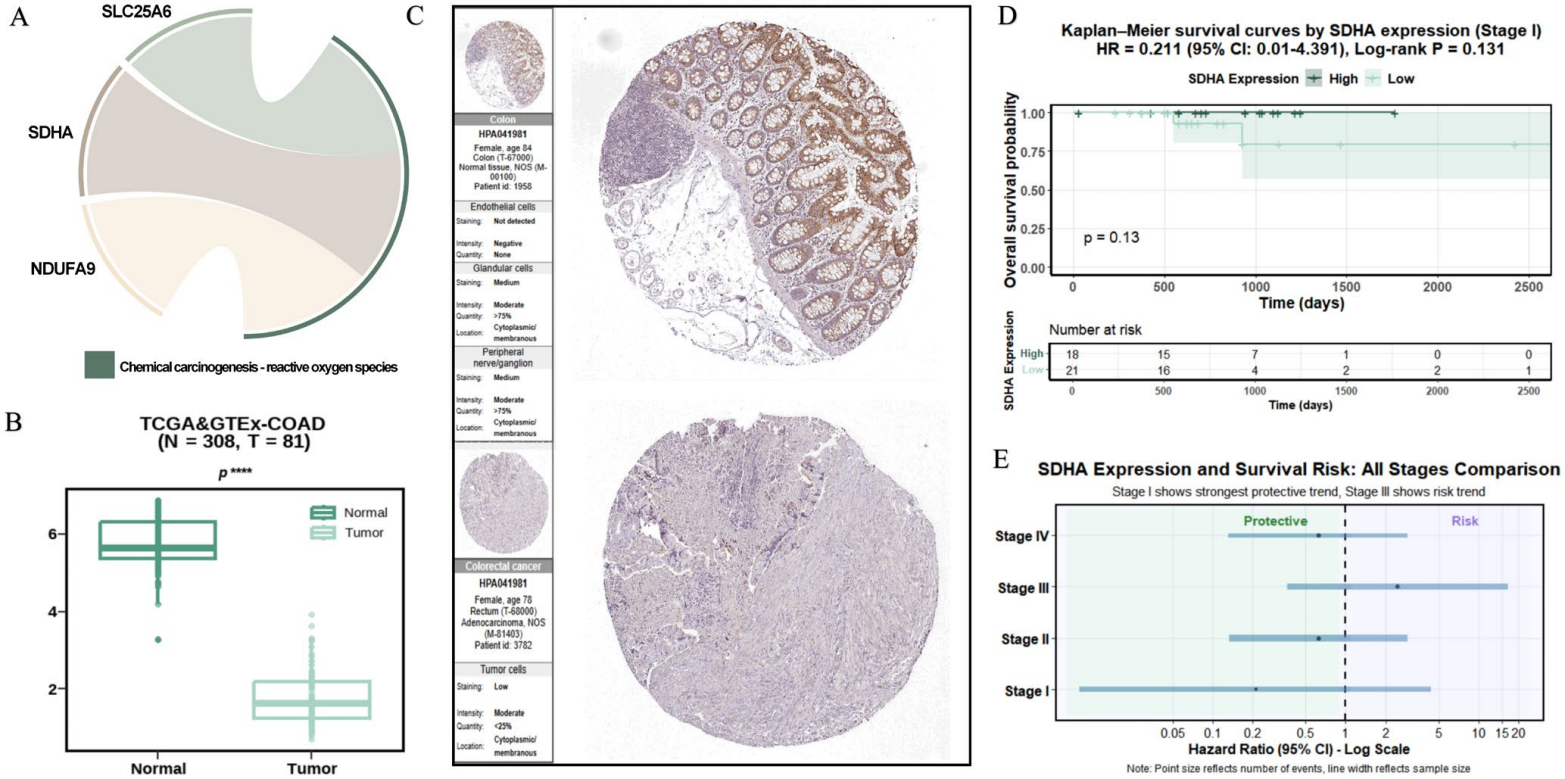
Multi‐dimensional validation of SDHA expression and relevance analysis. (A) KEGG enrichment analysis of SDHA in cancer‐related pathways. (B) SDHA mRNA expression in stage I COAD and matched normal tissues. (C) Immunohistochemical staining of SDHA in normal colonic epithelium and COAD cells (HPA database). Scale bar, 100 μm. (D) Kaplan–Meier survival analysis stratified by SDHA expression level in stage I COAD. (E) Forest plot of SDHA expression and survival risk across all COAD stages. Point size reflects number of events; line width reflects sample size. The number of patients and events (deaths) per stage were as follows: Stage I: *N* = 39, events = 2; Stage II: *N* = 76, events = 6; Stage III: *N* = 47, events = 4; Stage IV: *N* = 23, events = 6. HPA, Human Protein Atlas.

MD simulations revealed a stable and high‐affinity interaction between SDHA and CuI, supporting the selection of SDHA as the primary target for subsequent validation. This prioritization was further reinforced by pathway enrichment analysis, which implicated SDHA in ROS‐related signalling (Figure 5A). Consistent with this functional implication, transcriptomic analysis demonstrated that SDHA expression was significantly reduced in COAD tissues compared with normal controls (Figure 5B), indicating early disruption of mitochondrial metabolic homeostasis. This downregulation was corroborated at the protein level by immunohistochemical data from the Human Protein Atlas (HPA) database [47], which showed decreased SDHA expression in tumour tissues relative to normal colonic epithelium (Figure 5C).

Clinical survival analysis showed that higher SDHA expression in stage I patients exhibited a trend toward improved survival, although this association did not reach statistical significance (HR = 0.211, *p* = 0.131) (Figure 5D). Notably, stage‐stratified analysis revealed a dynamic shift in this association. The protective trend observed in stage I diminished in later stages and reversed toward a potential risk association in stage III (HR = 2.442) (Figure 5E; Figure S5).

Taken together, these findings suggest that the role of SDHA is dynamic and evolves during disease progression. This stage‐dependent pattern is consistent with the l‐DNB framework, which predicts increased network instability during early transitional phases. In this context, SDHA emerges as a central PB whose biological and clinical relevance is most pronounced at the early stage of COAD progression.

### 3.7 CuI Suppresses COAD Cell Proliferation and Modulates the SDHA/NF‐κB Axis

**FIGURE 6:**
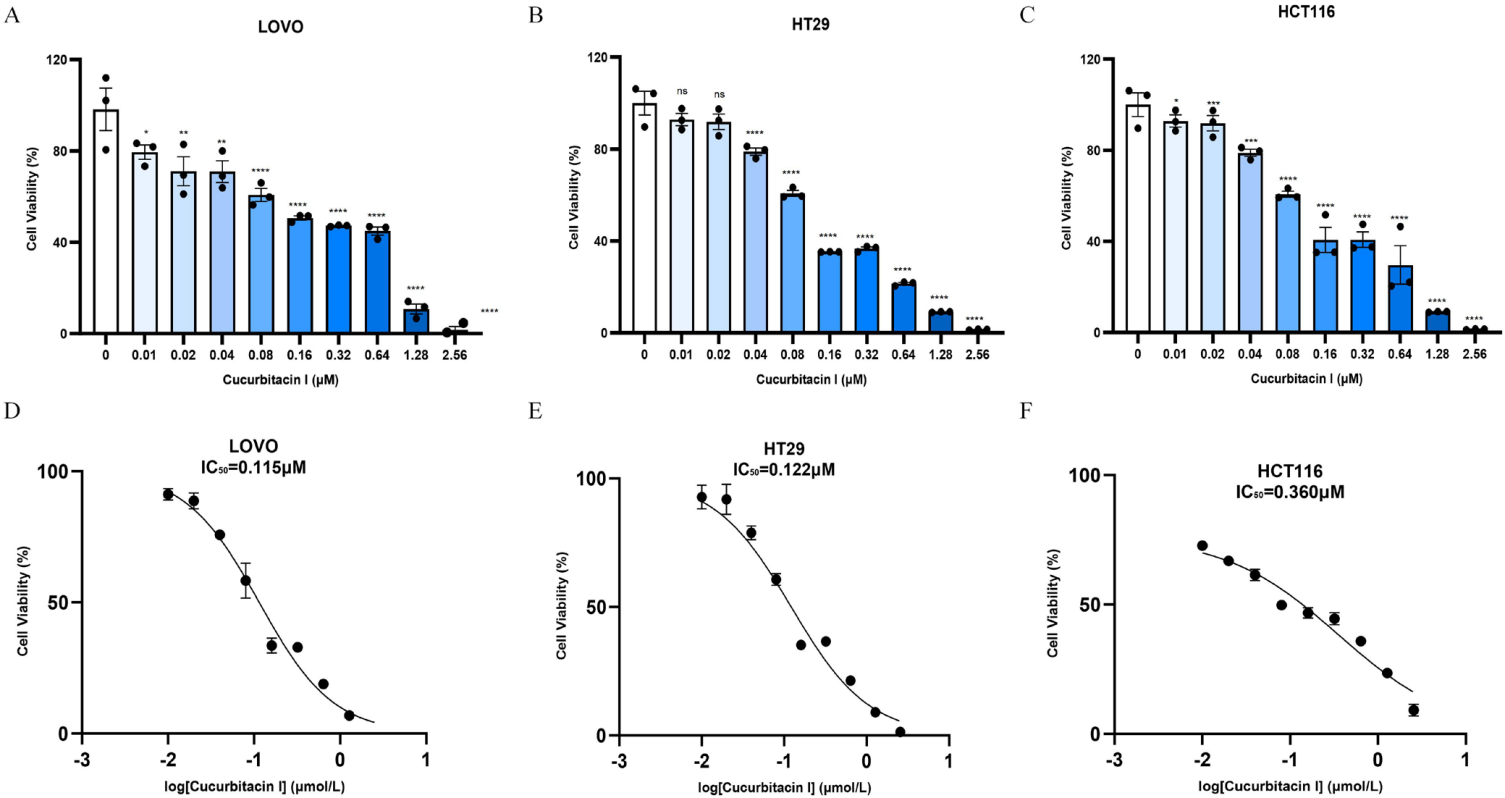
CuI inhibits the proliferation of COAD cells in a concentration‐dependent manner. (A‐C) CCK‐8 assay showing the survival rate of LOVO (A), HT29 (B) and HCT116 (C) cells treated with increasing concentrations of CuI for 48 h. The results indicate that CuI suppresses COAD cell proliferation in a concentration‐dependent manner. (D‐F) Doseresponse curves of CuI in LOVO (D), HT29 (E) and HCT116 (F) cells, with corresponding IC_50_ values calculated from the CCK‐8 assay. Data are presented as mean ± SEM, *n* = 3. One‐way ANOVA. *p < 0.05, **p < 0.01, ***p < 0.001, ****p < 0.0001 versus vehicle control.

To evaluate the therapeutic relevance of PB‐targeting components, we examined the effects of CuI on the proliferation of three COAD cell lines. CuI treatment produced a dose‐dependent inhibitory effect across all tested cells, with sub‐micromolar IC_50_ values observed in LOVO (0.115 μM), HT29 (0.122 μM) and HCT116 (0.360 μM) cells (Figure 6), indicating potent anti‐proliferative activity. Notably, sensitivity to CuI followed the order LOVO > HT29 > HCT116, which corresponded to their baseline SDHA expression levels (Figure S6), suggesting a potential association between SDHA status and cellular responsiveness.

**FIGURE 7:**
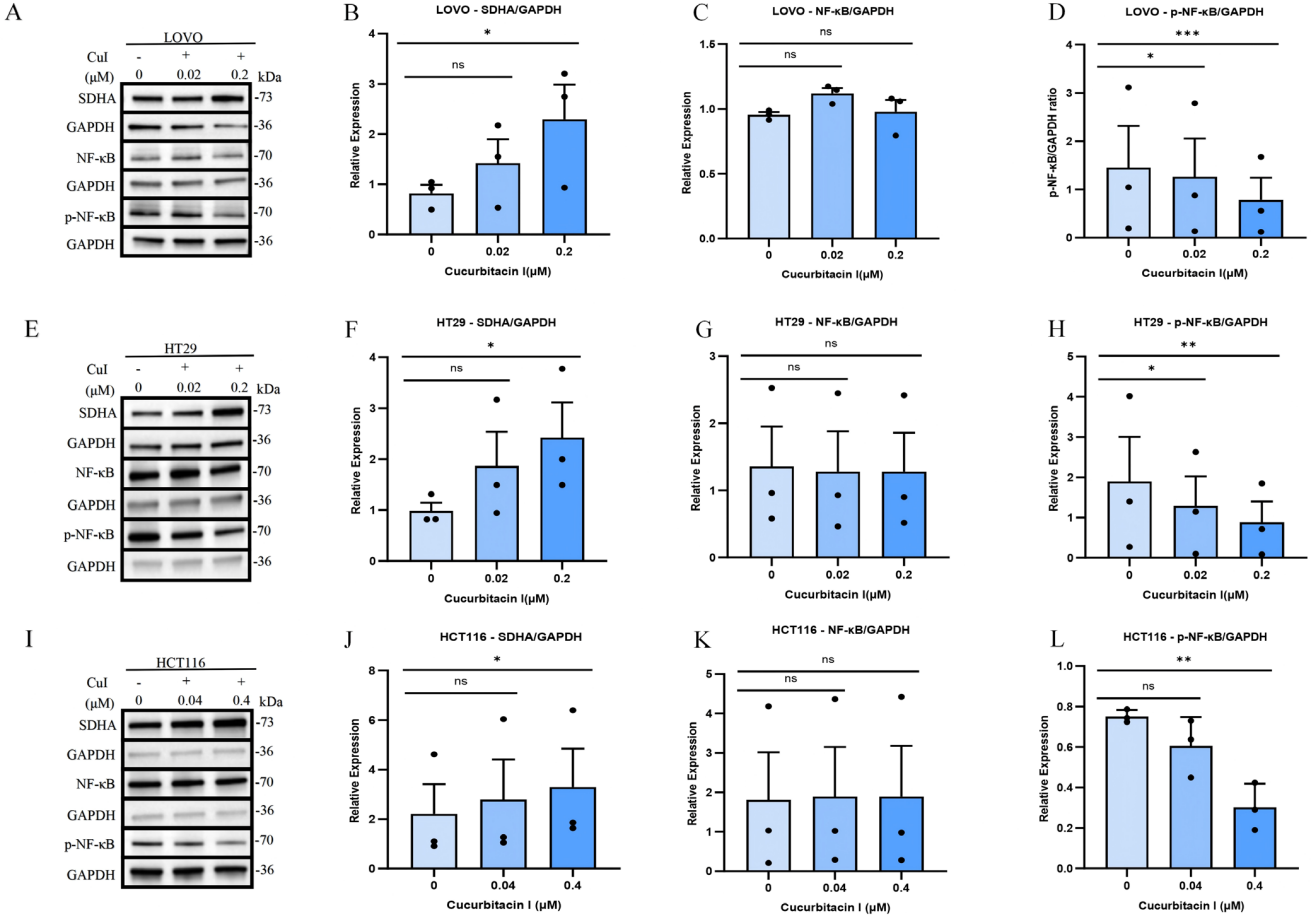
CuI upregulates SDHA and inhibits NF‐κB activation in COAD cells. (A, E, I) Representative Western blots of SDHA, total NF‐κB and p‐NF‐κB in LOVO (A), HT29 (E), and HCT116 (I) cells treated with increasing concentrations of CuI. (B, F, J) SDHA quantification showing significant dose‐dependent upregulation at IC₅₀ concentrations in all three cell lines (*p* < 0.05). (C, G, K) Total NF‐κB remained unchanged. (D, H, L) p‐NF‐κB was significantly decreased (*p* < 0.05 to 0.001). Data are presented as mean ± SEM from three independent experiments. Individual data points represent the results of each independent experiment (*n* = 3). One‐way ANOVA followed by Dunnett's test. *p < 0.05, **p < 0.01, ***p < 0.001 versus vehicle control.

Given that PBs enrichment analysis highlighted mitochondrial and ROS‐related pathways, and considering that NF‐κB signalling is a well‐established downstream effector of oxidative stress, we next investigated whether CuI influences the SDHA/NF‐κB axis in a concentration‐dependent manner. Western blot analysis further showed that CuI treatment significantly and dose‐dependently promoted the protein abundance of SDHA in all three cell lines (LOVO: 2.66 ± 0.46‐fold, *p* < 0.05; HT29: 2.38 ± 0.31‐fold, *p* < 0.05; HCT116: 1.64 ± 0.21‐fold, *p* < 0.05) and reduced p‐NF‐κB levels (LOVO: 0.57 ± 0.03‐fold, *p* < 0.001; HT29: 0.43 ± 0.06‐fold, *p* < 0.01; HCT116: 0.41 ± 0.10‐fold, *p* < 0.01) without affecting total NF‐κB expression (all *p* > 0.05) in all three cell lines (Figure 7; Figure S7). These dose–response relationships confirm the specificity of CuI's effects on the SDHA/NF‐κB axis. This coordinated regulation pattern suggests that CuI may suppress COAD cell proliferation partly through restoration of SDHA‐associated mitochondrial function and attenuation of downstream inflammatory signalling. The observed inverse relationship between SDHA abundance and NF‐κB activation is consistent with a model in which metabolic disruption promotes pro‐tumorigenic signalling cascades.

## 4 Discussion

This study establishes PBs as a systems‐level framework for identifying early transition risk in COAD. Unlike conventional diagnostic or prognostic biomarkers [48, 49], PBs capture dynamic molecular perturbations that precede phenotypic progression, thereby enabling risk stratification before irreversible clinical deterioration occurs. Given that COAD accounts for more than 90% of CRC cases and that stage I patients generally exhibit favourable survival outcomes after surgery [50], the ability to identify individuals at risk for rapid progression within this early stage has substantial clinical implications [51].

By applying the *l*‐DNB method, we identified a set of PBs that reflect network perturbations preceding phenotypic progression, suggesting that early‐stage tumour systems may pass through a critical transition phase characterized by maximal molecular fluctuation. Such transition signals provide a mechanistic basis for identifying individuals at risk for rapid progression before irreversible clinical deterioration occurs. Although some PBs were detected across multiple stages, this likely reflects conserved metabolic dysfunction rather than stage‐independent transition states, emphasizing the importance of interpreting PBs within a dynamic systems framework rather than as static gene lists.

Among the identified PBs, SDHA emerged as a pivotal hub, substantiated by convergent evidence spanning network topology, functional enrichment, expression transcriptomic profiling and prognostic association. Its reduced expression in tumour tissues [52], together with the favourable survival tendency observed in stage I patients with higher expression, suggests that SDHA may function as a context‐dependent metabolic regulator during early tumour evolution. The observed stage‐dependent prognostic pattern further implies that the biological relevance of SDHA shifts during disease progression, consistent with theoretical predictions that key regulatory nodes exert maximal influence near critical transition points. These findings highlight the importance of prioritizing biomarkers that reflect dynamic network properties rather than late‐stage correlations.

Building on PB‐guided target identification, we next explored whether these progression‐associated nodes could inform therapeutic discovery. Computational screening of components capable of reversing PB expression signatures identified CuI as a candidate molecule, and subsequent structural analyses suggested stable binding between CuI and several PB proteins, particularly SDHA. This integrative prioritization strategy links network‐level disease biology with component identification, providing a rational framework for identifying candidate therapeutics that target early transition mechanisms rather than late‐stage phenotypes.

Experimental validation demonstrated that CuI inhibits proliferation across multiple COAD cell lines while upregulating SDHA expression and reducing NF‐κB phosphorylation without altering total NF‐κB levels. This coordinated modulation indicates that CuI may attenuate inflammatory signalling via metabolic pathways. Because PB enrichment analysis highlights ROS‐related pathways and NF‐κB is a known redox‐responsive transcription factor [53, 54, 55, 56], these findings suggest that metabolic restoration may suppress pro‐tumorigenic signalling cascades. Specifically, SDHA is a core catalytic subunit of mitochondrial complex II (succinate dehydrogenase) that plays a central role in both the tricarboxylic acid cycle and the electron transport chain. Mitochondrial dysfunction resulting from reduced SDHA activity can lead to succinate accumulation and increased ROS production, both of which are established activators of the NF‐κB pathway [57]. Conversely, restoration of SDHA expression, as observed with CuI treatment, may restore mitochondrial electron flow, reduce oxidative stress and thereby attenuate NF‐κB phosphorylation. This is supported by prior studies showing that succinate dehydrogenase dysfunction promotes NF‐κB activation in cancer [58], and that metabolic intermediates serve as signalling molecules linking mitochondrial metabolism to inflammatory pathways [15]. However, direct measurement of ROS levels was not performed, and the mechanistic relationship between SDHA activity, oxidative stress and NF‐κB signalling requires further investigation through genetic and metabolic assays.

Although these findings support a potential link between metabolic regulation and inflammatory signalling, several limitations warrant consideration. Direct measurement of ROS levels was not performed, leaving the mechanistic relationship among SDHA activity, oxidative stress and NF‐κB signalling unresolved. The cohort size for early‐stage samples was relatively modest, which may reduce statistical power in survival analyses. Causal validation of SDHA function through genetic perturbation remains necessary. In vivo studies are also required to establish the therapeutic potential of CuI as well as its pharmacokinetic and safety characteristics. Addressing these issues will be essential for translating PB‐guided strategies into clinically applicable approaches.

Overall, this study demonstrates that monitoring dynamic molecular instability can reveal actionable biomarkers before overt clinical progression. By shifting emphasis from detecting established disease to anticipating progression risk, PB‐based strategies may provide a conceptual framework for earlier intervention and improved clinical management of COAD.

## 5 Conclusions

Integrating l‐DNB analysis with network pharmacology, this study identifies 20 PBs associated with stage I COAD and highlights SDHA as a core biomarker warranting further validation. Molecular docking and MD simulations identify CuI as a high‐affinity SDHA‐binding component, and cellular experiments support its anti‐tumour activity through modulation of the SDHA/NF‐κB axis. Although additional validation is required, this integrative framework establishes a mechanistically grounded strategy for early‐stage intervention and provides a conceptual paradigm for translating DNB into therapeutic discovery.

## Author Contributions

**Xunzhi Zhang:** validation. **Yue Lin:** methodology. **Danni Zhao:** writing – original draft, methodology, validation, visualization, software, formal analysis. **Wei Chen:** conceptualization, writing – review and editing, resources, funding acquisition, supervision. **Xinyu Tang:** software. **Pengmian Feng:** conceptualization, writing – review and editing. **Wenke Xiao:** software. **Zhixiang Wu:** methodology. **Qian Cheng:** investigation.

## Funding

This work was supported by the Natural Science Foundation of Sichuan Provience (No. 2024ZDZX0019).

## Ethics Statement

The authors have nothing to report.

## Consent

All authors reviewed and approved the final manuscript.

## Conflicts of Interest

The authors declare no conflicts of interest.

## Supporting information


**Figure S1:** Visualization of batch‐corrected expression and pathological stages DEGs in COAD.
**Figure S2:** Overlap analysis of DEGs in COAD stages I‐IV.
**Figure S3:** Sensitivity analysis for the selection of *k* = 20 as the DNB module size.
**Figure S4:** MD simulation results for the top five PBs with CuI.
**Figure S5:** SDHA expression and survival outcomes in stage II‐IV COAD.
**Figure S6:** Validation of SDHA expression in LOVO, HCT116 and HT29 cells.
**Figure S7:** CuI dose‐dependently upregulates SDHA and inhibits p‐NF‐κB in LOVO cells.
**Table S1:** Sample stage information for the COAD datasets.
**Table S2:** Detailed information of $I_{s}$ for top 20 candidate PBs and $I_{\mathrm{DNB}}$ across different pathological stages of COAD.
**Table S3:** Top 50 components identified by L1000CDS^2^ as potential reversers of the COAD transcriptional signature.

## Data Availability

The datasets analysed in this study are publicly available and mentioned in the Materials and Methods section.
