# Supplementary material for: Therapeutic Potential of Cucurbitacin I in Colon Adenocarcinoma Is Mediated by Modulation of SDHA Expression
Source: J Cell Mol Med. 2026 Jun 9;30(11):e71231. doi: 10.1111/jcmm.71231 (PMC13249804; doi:10.1111/jcmm.71231)
Supplement: Supplementary file 1 — Figure S1: Visualization of batch‐corrected expression and pathological stages DEGs in COAD. Figure S2: Overlap analysis of DEGs in COAD stages I‐IV. Figure S3: Sensitivity analysis for the selection of k = 20 as the DNB module size. Figure S4: MD simulation results for the top five PBs with CuI. Figure S5: SDHA expression and survival outcomes in stage II‐IV COAD. Figure S6: Validation of SDHA expression in LOVO, HCT116 and HT29 cells. Figure S7: CuI dose‐dependently upregulates SDHA and inhibits p‐NF‐κB in LOVO cells. Table S1: Sample stage information for the COAD datasets. Table S2: Detailed information of Is for top 20 candidate PBs and IDNB across different pathological stages of COAD. Table S3: Top 50 components identified by L1000CDS2 as potential reversers of the COAD transcriptional signature. [file JCMM-30-e71231-s001.docx]

**Supplementary Material**

**Supplementary Methods**

**Detailed procedure of the landscape dynamic network biomarker (*l*-DNB) method.**

The *l*-DNB method for progression biomarkers (PBs) identification and critical state determination in colon adenocarcinoma (COAD) involves three steps.

Step 1 involves constructing the single-sample network (SSN) for a given normal sample using Equations (1) and (2), based on the difference in the pearson correlation coefficient ($\text{PCC}$) values between the two genes $\text{x}$ and $\text{y}$.

|  | $\text{PCC}_{\text{n}}\text{(}\text{x}\text{,}\text{y}\text{)=}\frac{\sum_{\text{i}\text{=1}}^{\text{n}} \text{(}\text{x}_{\text{i}}\text{-}\bar{\text{x}}\text{)(}\text{y}_{\text{i}}\text{-}\bar{\text{y}}\text{)}}{\sqrt{\sum_{\text{i}\text{=1}}^{\text{n}} {\text{(}\text{x}_{\text{i}}\text{-}\bar{\text{x}}\text{)}}^{\text{2}}\sum_{\text{i}\text{=1}}^{\text{n}} {\text{(}\text{y}_{\text{i}}\text{-}\bar{\text{y}}\text{)}}^{\text{2}}}}$ | (1) |
| --- | --- | --- |

The variables $\text{x}_{\text{i}}$ and $\text{y}_{\text{i}}$ represent the expression values of genes $\text{x}$ and $\text{y}$ in the $\text{n}$ normal samples. Meanwhile, $\bar{\text{x}}$ and $\bar{\text{y}}$ denote the average expression values of genes $\text{x}$ and $\text{y}$ across the normal samples. Additionally, $\text{PCC}_{\text{n}}\left( \text{x}\text{,}\text{y} \right)$ indicates the correlation between genes $\text{x}$ and $\text{y}$ in the $\text{n}$ normal samples. After incorporating the new COAD sample (sample $\text{d}$) into the set of normal samples, the total number of samples increases to $\text{n}\text{+1}$ (comprising $\text{n}$ reference samples and one additional sample $\text{d}$).


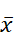


|  | $\text{sPCC}_{\text{n}}\left( \text{x}\text{,}\text{y} \right)\text{=}\text{PCC}_{\text{n}\text{+1}}\left( \text{x}\text{,}\text{y} \right)\text{-}\text{PCC}_{\text{n}}\text{(}\text{x}\text{,}\text{y}\text{)}$ | (2) |
| --- | --- | --- |

The new $\text{PCC}$, $\text{PCC}_{\text{n}\text{+1}}\left( \text{x}\text{,}\text{y} \right)$ calculated using Equation (2), represents the relationship between the two genes $\text{x}$ and $\text{y}$. The disparity between $\text{PCC}_{\text{n}\text{+1}}$ and $\text{PCC}_{\text{n}}$ results from incorporating the sample $\text{d}$ among the normal samples, highlighting the correlation difference between the sample $\text{d}$ and the $\text{n}$ normal samples. These steps are essential for constructing the SSN for sample $\text{d}$.

Step 2 (a) Computing the deviation of gene $\text{x}$’s expression value in an individual sample compared to the expression values in $\text{n}$ normal samples, known as single-sample expression deviation ($\text{sED}$).

|  | $\text{sED}\left( \text{x}_{\text{d}} \right)\text{=}\left\vert\text{x}_{\text{d}}\text{-}\bar{\text{x}} \right\vert$ | (3) |
| --- | --- | --- |
|  | $\bar{\text{x}}\text{=}\frac{\text{1}}{\text{n}}\sum_{\text{i}\text{=1}}^{\text{n}} \text{x}_{\text{i}}$ | (4) |

The expression level of gene $\text{x}$ in sample $\text{d}$ is denoted as $\text{x}_{\text{d}}$. $\bar{\text{x}}$ is the average expression level of gene $\text{x}$ across $\text{n}$ normal samples. $\text{sED}_{\text{in}}$ is the mean expression deviation of all $\left( \text{1+}\text{n}_{\text{xd}} \right)$ genes within the local module of gene $\text{x}$ in sample $\text{d}$, relative to the normal samples.

|  | $\text{sED}_{\text{in}}\text{=}\frac{\text{1}}{\text{1+}\text{n}_{\text{x}_{\text{d}}}}\left[ \text{sED}\left( \text{x}_{\text{d}} \right)\text{+}\sum_{\text{y}_{\text{d}}\text{∈}\text{N}_{\text{x}_{\text{d}}}} \text{sED}\text{(}\text{y}_{\text{d}}\text{)} \right]$ | (5) |
| --- | --- | --- |

The local module comprises gene $\text{x}$ and its first-order neighboring genes. These neighboring genes are represented by $\text{N}_{\text{xd}}$, where $\text{N}_{\text{xd}}$ refers to the gene set containing $\text{n}_{\text{xd}}$ genes.

(b) The local module’s $\text{sPCC}_{\text{in}}$ is calculated as $\text{sPCC}_{\text{n}}$ between gene $\text{x}$ and its first-order neighboring genes $\text{N}_{\text{xd}}$ in the SSN.


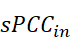


|  | $\text{sPCC}_{\text{in}}\text{=}\frac{\text{1}}{\text{n}_{\text{x}_{\text{d}}}}\sum_{\text{y}_{\text{d}}\text{∈}\text{N}_{\text{x}_{\text{d}}}} \left\vert\text{sPCC}_{\text{n}}\text{(}\text{x}_{\text{d}}\text{,}\text{y}_{\text{d}}\text{)} \right\vert$ | (6) |
| --- | --- | --- |

When the correlation of the newly added sample $\text{PCC}_{\text{n}\text{+1}}$ increases, $\text{sPCC}_{\text{n}}\text{(}\text{x}_{\text{d}}\text{,}\text{y}_{\text{d}}$) also increases, as demonstrated in Equations (2) and (6). This results in a sharp rise in $\text{sPCC}_{\text{in}}$. The $\text{sPCC}_{\text{out}}$ value represents the average of $\text{s}\text{PCC}_{\text{n}}$ between the first-order neighboring genes $\text{N}_{\text{xd}}$ and the second-order neighboring genes $\text{M}_{\text{x}_{\text{d}}}$, within the local module of gene $\text{x}$.


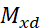


|  | $\text{sPCC}_{\text{out}}\text{=}\frac{\text{1}}{\text{n}_{\text{x}_{\text{d}}}\text{m}_{\text{x}_{\text{d}}}}\sum_{\text{x}_{\text{d}}\text{∈}\text{N}_{\text{x}_{\text{d}}}\text{,}\text{y}_{\text{d}}\text{∈}\text{M}_{\text{x}_{\text{d}}}} \left\vert\text{sPCC}_{\text{n}}\text{(}\text{x}_{\text{d}}\text{,}\text{y}_{\text{d}}\text{)} \right\vert$ | (7) |
| --- | --- | --- |

The collection of second-order neighboring genes of gene x is represented by $\text{M}_{\text{x}_{\text{d}}}$, where $\text{m}_{\text{x}_{\text{d}}}$ denotes the number of genes in $\text{M}_{\text{x}_{\text{d}}}$.


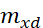


(c) The local dynamic network biomarker score ($\text{I}_{\text{s}}$) is derived from the local modules of gene $\text{x}$ within a single sample, serving as the criticality score for that gene.


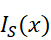


|  | $\text{I}_{\text{s}}\text{(}\text{x}\text{)=}\text{sED}_{\text{in}}\frac{\text{sPCC}_{\text{in}}}{\text{sPCC}_{\text{out}}}$ | (8) |
| --- | --- | --- |

Step 3 involves identifying the dynamic network biomarker module and obtaining the global dynamic network biomarker score ($\text{I}_{\text{DNB}}$) for a single sample. The appearance of the dynamic network biomarker module signifies that the system is shifting from a normal state to a diseased state.

|  | $\text{I}_{\text{DNB}}\text{=}\sum_{\text{x}\text{=1}}^{\text{k}} \frac{\text{I}_{\text{s}}\text{(}\text{x}\text{)}}{\text{k}}$ | (9) |
| --- | --- | --- |

The top $\text{k}$ genes (*k* = 20 in this study) with the highest $\text{I}_{\text{s}}$ were selected as potential dynamic network biomarker members. **The selection of** *k*=20 **was based on two complementary analyses: (i) cumulative score analysis demonstrated that the top 20 genes accounted for 52.7% of the total cumulative criticality score of the top 50 genes, and (ii) marginal gain analysis revealed a sharp decline in individual criticality scores after** *k*=20**, with the mean score dropping from 19.93 (top 20) to 11.91 (genes 21~50), representing a 40.2% decrease (Supplementary Figure 3).** The $\text{I}_{\text{DNB}}$ was then calculated as the mean of these 20 $\text{I}_{\text{s}}$. The pathological stage exhibiting the highest $\text{I}_{\text{DNB}}$ was identified as the critical state. The 20 genes with the highest $\text{I}_{\text{s}}$ in stage I samples were designated as PBs.


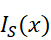

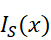


**Supplementary Figures**

**
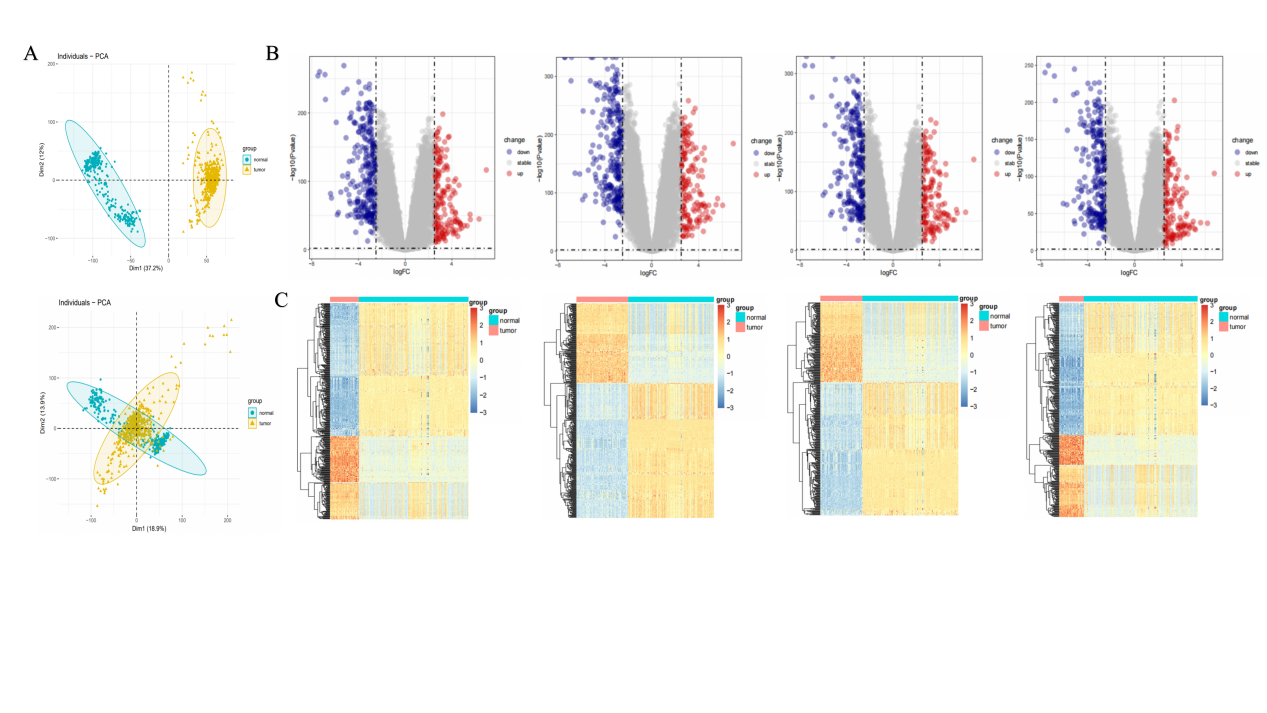
**

**Supplementary Fig. 1.** Visualization of batch-corrected expression and pathological stages DEGs in COAD. (A) PCA of TCGA-COAD and GTEx normal colon tissue samples before (up panel) and after (down panel) batch correction using the normalizeBetweenArrays function from the limma R package, confirming the removal of systematic inter-database differences. (B) The volcano plot displays DEGs across COAD stages I-IV, with red indicating upregulation, blue indicating downregulation, and grey indicating no significant difference (|log₂FC| > 2.5 and *P* < 0.01). (C) Heatmap of DEGs across COAD stages I-IV. Genes are arranged in rows and samples in columns. The color scale represents normalized expression values. Abbreviations: DEGs, differentially expressed genes; COAD, colon adenocarcinoma; PCA, principal component analysis; TCGA, The Cancer Genome Atlas; GTEx, Genotype-Tissue Expression; FC, fold change.


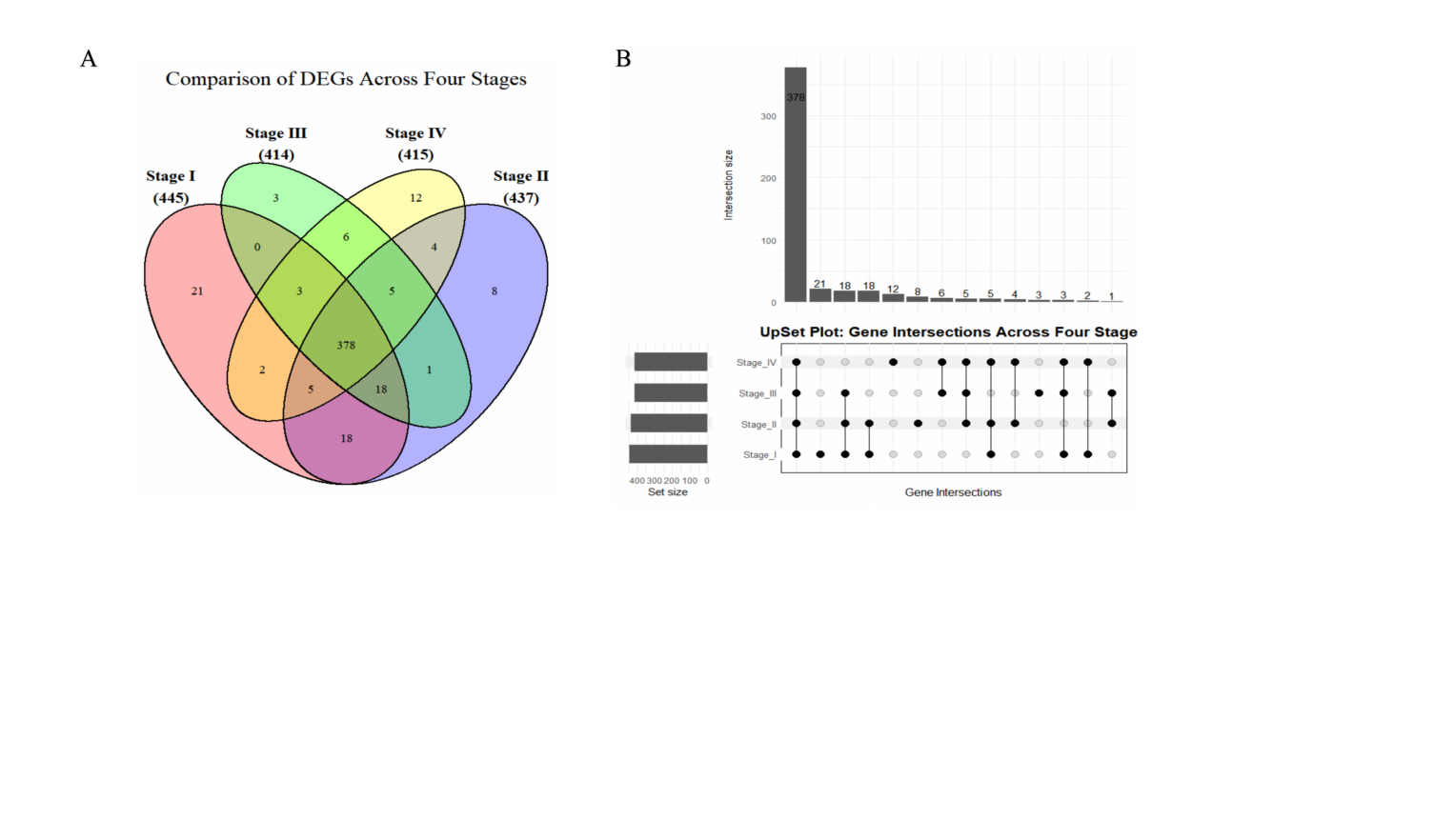


**Supplementary Fig. 2.** Overlap analysis of DEGs in COAD stages I-IV. (A) Venn diagram showing DEG overlap across stages I-IV. Values indicate DEGs unique to or shared among stages. A core set of 378 DEGs was common to all four stages, suggesting persistently dysregulated genes throughout progression. (B) UpSet plot visualizing intersection sizes and compositions. Bars represent intersection sizes; the matrix indicates involved stages. The leftmost bar confirms the core DEGs shared across all stages. Other bars show substantially smaller overlaps for different stage combinations, indicating comparable stagewise DEG counts with a high proportion of core overlapping genes.

**Supplementary Fig. 3.** Sensitivity analysis for the selection of *k* = 20 as the DNB module size. (A) Cumulative criticality score analysis. The cumulative criticality score of the top *k* genes is presented as a percentage of the total score accumulated by the top 50 genes (after which the marginal gain reaches a plateau). The top 20 genes contributed 52.7% of the total cumulative score. (B) Marginal gain analysis. Individual gene criticality scores (*Iₛ*) are plotted in descending order. The dashed vertical line at *k* = 20 marks the inflection point. Notably, the mean Iₛ score drops by 40.2% from 19.93 for the top 20 genes to 11.91 for genes ranked 21~50. These analyses demonstrate that selecting *k* = 20 can capture the dominant critical module while excluding low-signal noise.


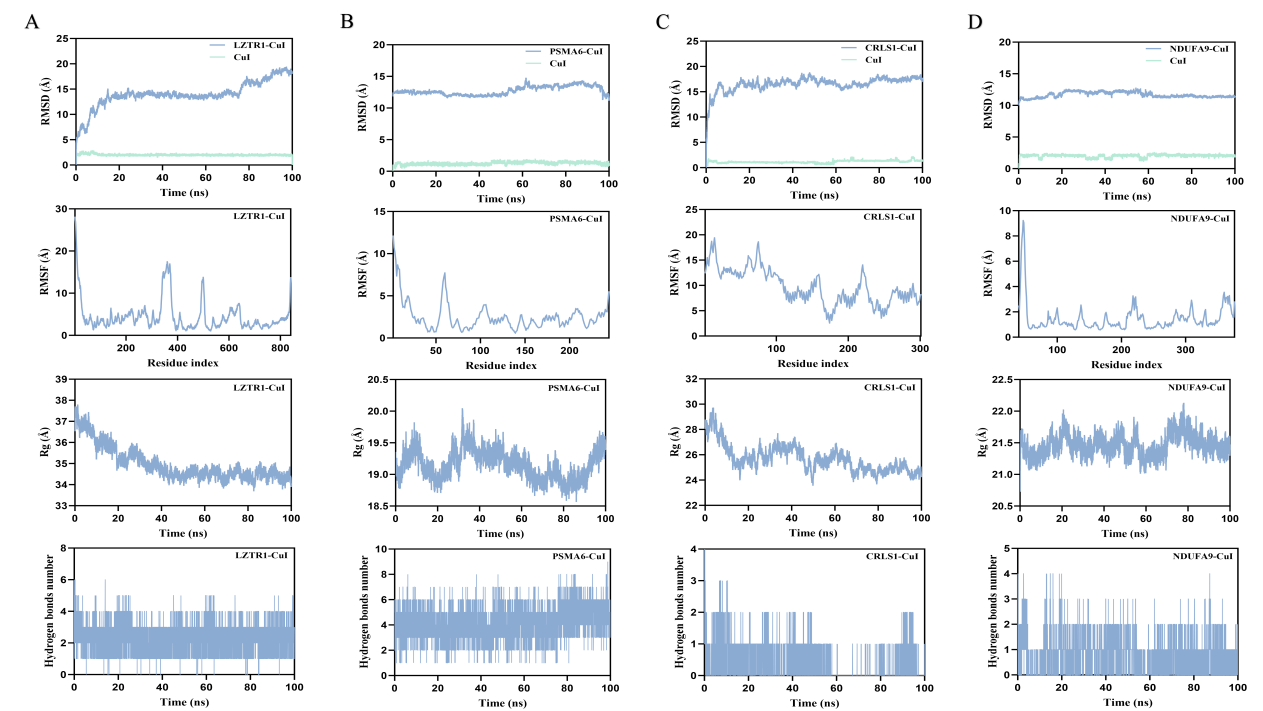


**Supplementary Fig. 4.** MD simulation results for the top five PBs with CuI. (A-E) Visualization of 100 ns MD results for CuI complexes with LZTR1, CRLS1, PSMA6, and NDUFA9, respectively. The first column illustrates the RMSD results, the second column depicts the RMSF, the third column indicates overall compactness through Rg, and the fourth column provides hydrogen bond count statistics. Abbreviations: MD, molecular dynamics; PBs, progression biomarkers; CuI, cucurbitacin I; LZTR1, leucine zipper transcription regulator-like 1; CRLS1, cardiolipin synthase 1; PSMA6, proteasome subunit α 6; NDUFA9, NADH: ubiquinone oxidoreductase subunit A9; RMSD, root mean square deviation; RMSF, root mean square fluctuation; Rg, radius of gyration.

**
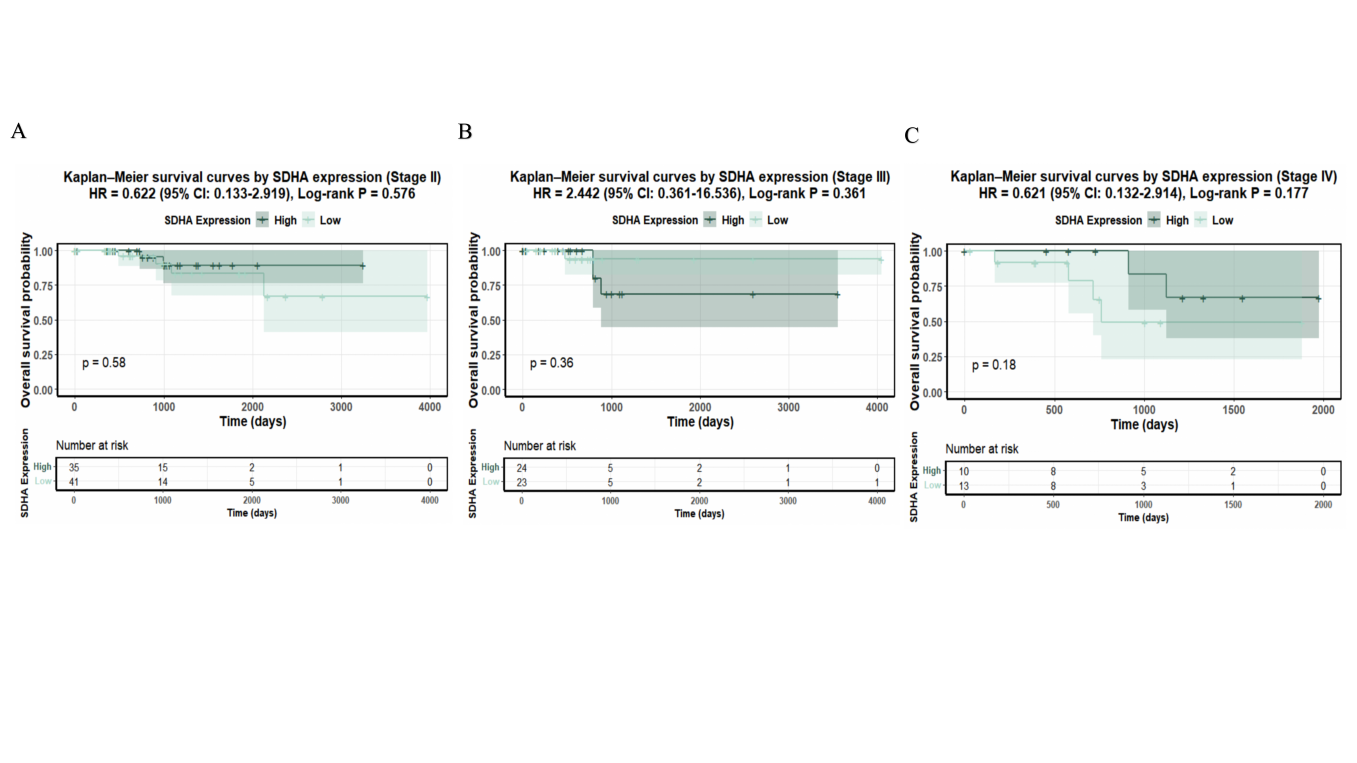
**

**Supplementary Fig. 5.** SDHA expression and survival outcomes in stage II-IV COAD. Kaplan-Meier survival curves comparing high vs. low SDHA expression in (A) Stage II (n=76), (B) Stage III (n=47), and (C) Stage IV (n=23) COAD patients. Stage II: HR = 0.622 (95% CI: 0.133-2.919), *P* = 0.576; stage III: HR = 2.442 (95% CI: 0.361-16.536), *P* = 0.361; stage IV: HR = 0.621 (95% CI: 0.132-2.914), *P* = 0.177. No comparisons reached statistical significance, likely due to limited sample sizes and event numbers. Shaded areas represent 95% CI. Abbreviations: SDHA, succinate dehydrogenase complex flavoprotein subunit A; HR, hazard ratio; CI, confidence interval.


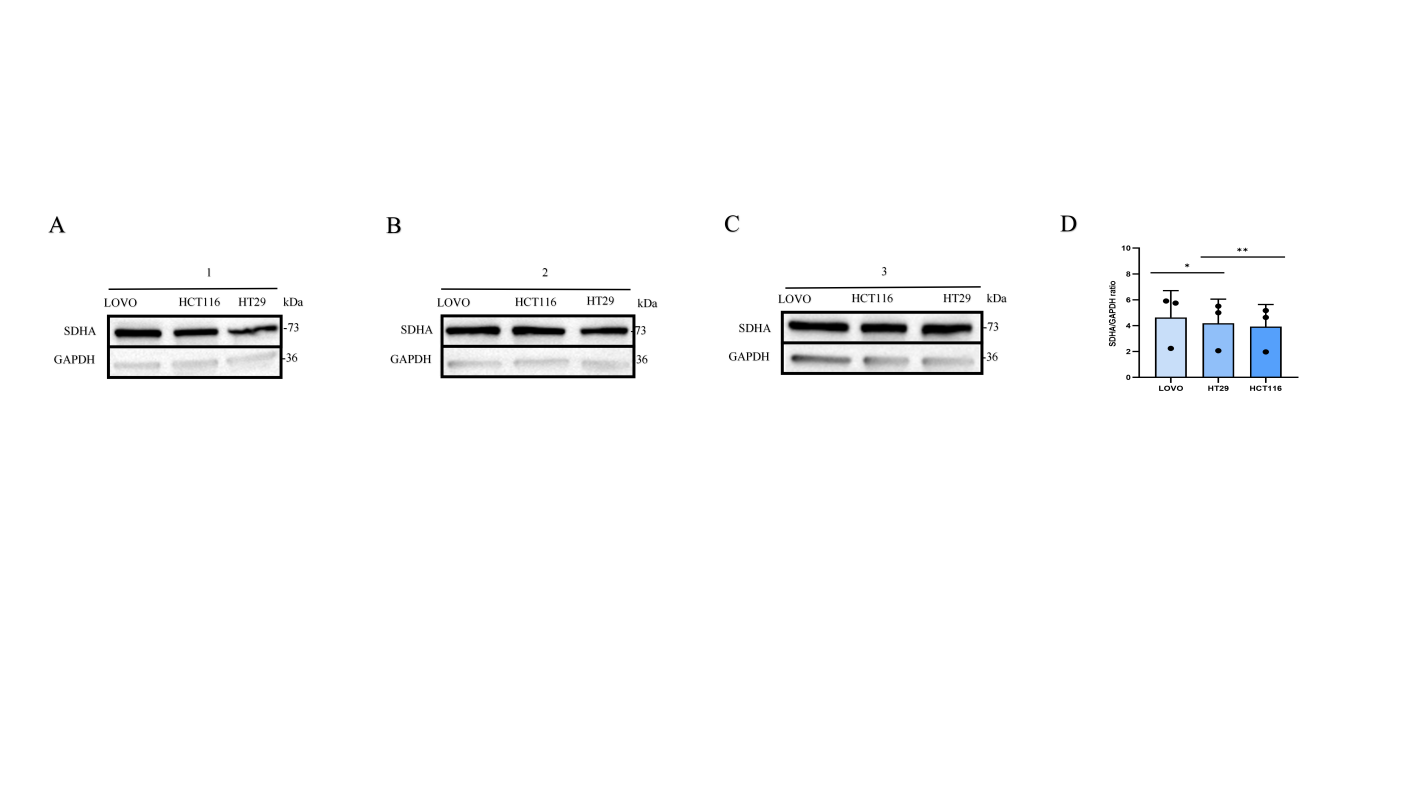


**Supplementary Fig. 6.** Validation of SDHA expression in LOVO, HCT116, and HT29 cells. (A-C) Three independent representative Western blots showing baseline SDHA expression in LOVO, HCT116, and HT29 cells prior to CuI treatment. GAPDH was used as a loading control. (D) Quantification of SDHA protein levels normalized to GAPDH. Data are presented as mean ± SEM from three independent experiments. One-way ANOVA followed by Dunnett’s test. **P* < 0.05, ***P* < 0.01 vs. LOVO cells.

**
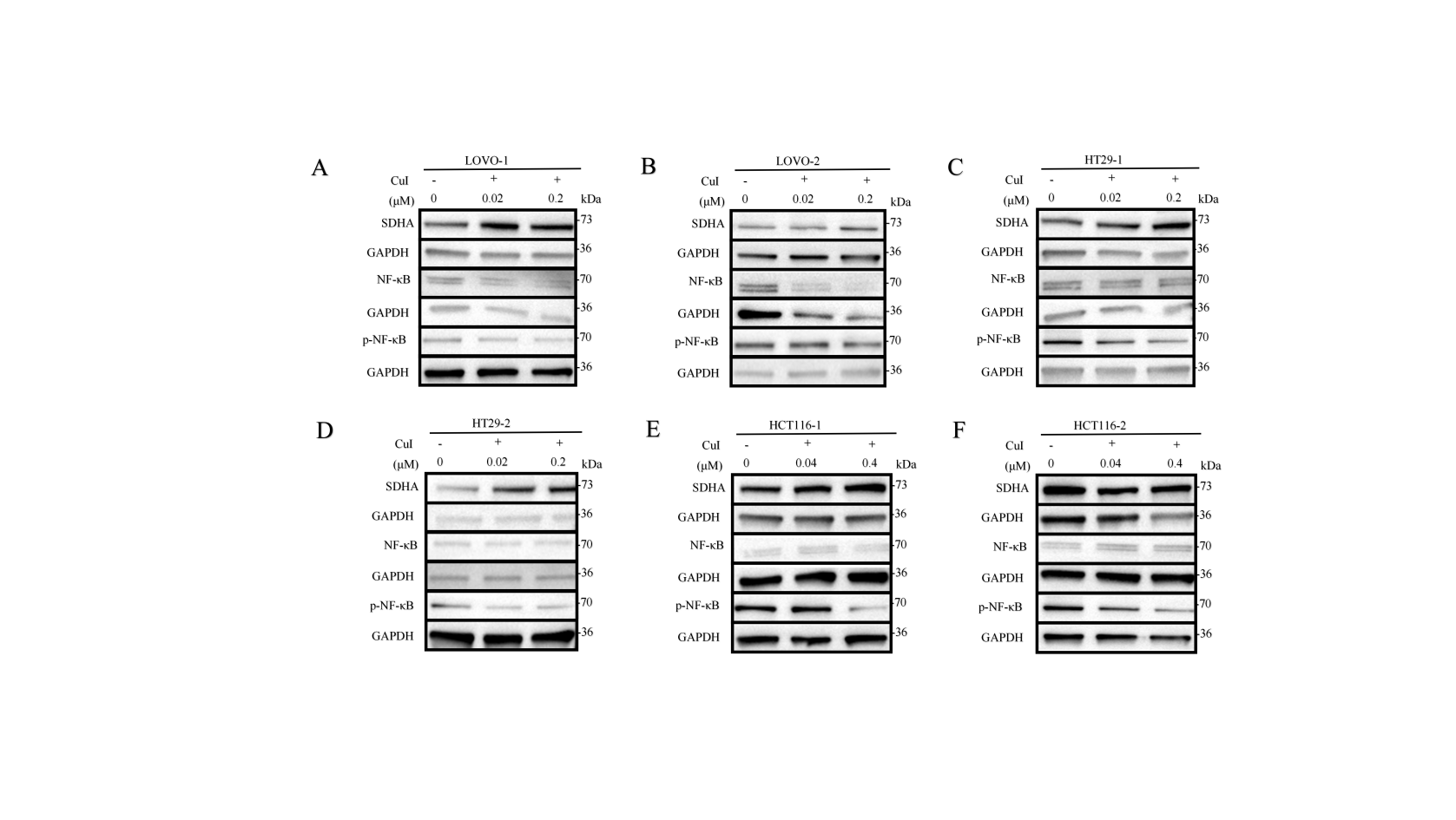
**

**Supplementary Fig. 7.** CuI dose-dependently upregulates SDHA and inhibits p-NF-κB in LOVO cells. (A-F) Western blot analysis of SDHA, total NF-κB, and p-NF-κB in LOVO (A, B), HT29 (C, D), and HCT116 (E, F) cells treated with CuI at the indicated concentrations: LOVO and HT29 cells received 0, 0.02, and 0.2 μM; HCT116 cells received 0, 0.04, and 0.4 μM, based on their respective IC₅₀ values. GAPDH was used as a loading control.

**Supplementary Tables**

**Supplementary Table S1.** Sample stage information for the COAD datasets.

| Stage | Upregulated | Downregulated | DEGs number | Sample number | |
| --- | --- | --- | --- | --- | --- |
| Stage I | 169 | 276 | 445 | 81 | |
| Stage II | 162 | 275 | 437 | 187 | |
| Stage III | 155 | 259 | 414 | 133 | |
| Stage IV | 156 | 259 | 415 | 67 | |
| Total COAD samples (TCGA) | | | | | 468 |
| Normal samples (GTEx) | | | | | 308 |

Abbreviations: COAD, colon adenocarcinoma; DEG, differentially expressed gene; TCGA, the cancer genome atlas; GTEx, genotype-tissue expression.

**Supplementary Table S2.** Detailed information of $\text{I}_{\text{s}}$ for top 20 candidate PBs (screened by the *l*-DNB method) and $\text{I}_{\text{DNB}}$ across different pathological stages of COAD.

| Stage | Rank | Gene symbol | $\text{I}_{\text{s}}$ |
| --- | --- | --- | --- |
| I | 1 | PERP | 36.11 |
| I | 2 | SLC25A6 | 28.32 |
| I | 3 | CRLS1 | 27.80 |
| I | 4 | CSE1L | 25.06 |
| I | 5 | EMC6 | 23.44 |
| I | 6 | S100A11 | 22.42 |
| I | 7 | WDR27 | 22.02 |
| I | 8 | PDE4DIP | 20.85 |
| I | 9 | MRPL2 | 19.65 |
| I | 10 | LRCH4 | 19.12 |
| I | 11 | SDHA | 18.31 |
| I | 12 | CNPY2 | 16.69 |
| I | 13 | PSMA6 | 16.08 |
| I | 14 | CCZ1B | 15.82 |
| I | 15 | CMC2 | 14.99 |
| I | 16 | IRF9 | 14.91 |
| I | 17 | TTLL3 | 14.58 |
| I | 18 | LZTR1 | 14.50 |
| **Supplementary Table S2 (continued)** | | | |
| Stage | Rank | Gene symbol | $\text{I}_{\text{s}}$ |
| I | 19 | RPS10 | 14.01 |
| I | 20 | NDUFA9 | 13.91 |
| I | | $\text{I}_{\text{DNB}}$ | 19.93 |
| II | 1 | CRLS1 | 27.78 |
| II | 2 | CSE1L | 26.92 |
| II | 3 | EMC6 | 24.66 |
| II | 4 | CCZ1B | 23.69 |
| II | 5 | WDR27 | 23.22 |
| II | 6 | PERP | 23.21 |
| II | 7 | S100A11 | 23.15 |
| II | 8 | MRPL2 | 19.48 |
| II | 9 | FTL | 18.10 |
| II | 10 | SDHA | 18.07 |
| II | 11 | IRF9 | 16.82 |
| II | 12 | PSMA6 | 16.68 |
| II | 13 | LRCH4 | 16.56 |
| II | 14 | TTLL3 | 14.84 |
| II | 15 | PET100 | 14.45 |
| II | 16 | RFC3 | 14.34 |
| II | 17 | CNPY2 | 14.15 |
| **Supplementary Table S2 (continued)** | | | |
| Stage | Rank | Gene symbol | $\text{I}_{\text{s}}$ |
| II | 18 | LZTR1 | 14.01 |
| II | 19 | CHPF | 13.60 |
| II | 20 | EPB41L3 | 13.41 |
| II | | $\text{I}_{\text{DNB}}$ | 18.86 |
| III | 1 | CRLS1 | 27.30 |
| III | 2 | S100A11 | 26.81 |
| III | 3 | EMC6 | 24.49 |
| III | 4 | WDR27 | 23.60 |
| III | 5 | MRPL2 | 22.24 |
| III | 6 | CSE1L | 19.88 |
| III | 7 | PERP | 19.678 |
| III | 8 | SDHA | 18.93 |
| III | 9 | PDE4DIP | 18.62 |
| III | 10 | CCZ1B | 18.06 |
| III | 11 | CNPY2 | 16.40 |
| III | 12 | CMC2 | 16.25 |
| III | 13 | FTL | 16.00 |
| III | 14 | PET100 | 16.00 |
| III | 15 | PSMA6 | 15.64 |
| III | 16 | NDUFA9 | 15.61 |
| **Supplementary Table S2 (continued)** | | | |
| Stage | Rank | Gene symbol | $\text{I}_{\text{s}}$ |
| III | 17 | ZFPL1 | 14.70 |
| III | 18 | IRF9 | 14.60 |
| III | 19 | LRCH4 | 14.26 |
| III | 20 | LZTR1 | 13.98 |
| III | | $\text{I}_{\text{DNB}}$ | 18.65 |
| IV | 1 | CSE1L | 34.49 |
| IV | 2 | POMP | 34.12 |
| IV | 3 | EMC6 | 25.18 |
| IV | 4 | CRLS1 | 24.88 |
| IV | 5 | MRPL2 | 21.23 |
| IV | 6 | WDR27 | 20.63 |
| IV | 7 | PERP | 19.34 |
| IV | 8 | PDE4DIP | 19.31 |
| IV | 9 | SDHA | 19.25 |
| IV | 10 | LRCH4 | 16.49 |
| IV | 11 | S100A11 | 16.42 |
| IV | 12 | CCZ1B | 16.25 |
| IV | 13 | PET100 | 16.16 |
| IV | 14 | RFC3 | 16.09 |
| IV | 15 | CMC2 | 15.46 |
| **Supplementary Table S2 (continued)** | | | |
| Stage | Rank | Gene symbol | $\text{I}_{\text{s}}$ |
| IV | 16 | PSMA6 | 15.26 |
| IV | 17 | CNPY2 | 15.25 |
| IV | 18 | IRF9 | 15.21 |
| IV | 19 | TTLL3 | 15.17 |
| IV | 20 | FTL | 15.17 |
| IV | | $\text{I}_{\text{DNB}}$ | 19.57 |

Note: $\text{I}_{\text{DNB}}$ for each stage was calculated as the mean of the top 20 $\text{I}_{\text{s}}$ according to the *l*-DNB methodology. The 20 candidate PBs exhibited the highest $\text{I}_{\text{s}}$ in stage I and were selected as PBs for progression risk stratification.

Abbreviations: $\text{I}_{\text{s}}$, local dynamic network biomarker score; PBs, progression biomarkers; $\text{I}_{\text{DNB}}$, global dynamic network biomarker score; *l*-DNB, landscape dynamic network biomarker.

**Supplementary Table S3.** Top 50 components identified by L1000CDS^2^ as potential reversers of the COAD transcriptional signature.

| Rank | score | Perturbation | Cell line | Dose | Time |
| --- | --- | --- | --- | --- | --- |
| 1 | 0.2667 | TG101348 | A375 | 11.10μM | 24 h |
| 2 | 0.2667 | PLX-4032 | A375 | 10.0μM | 24 h |
| 3 | 0.2000 | Rottlerin | PC3 | 10.0μM | 24 h |
| 4 | 0.2000 | geldanamycin | HCC515 | 10.0μM | 6 h |
| 5 | 0.2000 | PP-110 | A375 | 22.2μM | 24 h |
| 6 | 0.2000 | KU 0060648 trihydrochloride | A375 | 10.0μM | 24 h |
| 7 | 0.2000 | BMS-536924 | A375 | 11.1μM | 24 h |
| 8 | 0.2000 | 2858522 | HT29 | 10.0μM | 24 h |
| 9 | 0.2000 | L-690,488 | HT29 | 10.0μM | 24 h |
| 10 | 0.2000 | cucurbitacin I | PC3 | 10.0μM | 24 h |
| 11 | 0.2000 | COT-10b | PC3 | 44.4μM | 24 h |
| 12 | 0.2000 | BRD-K43620258 | PC3 | 80.0μM | 24 h |
| 13 | 0.2000 | Tyrphostin AG 1478 | PC3 | 56.78μM | 24 h |
| 14 | 0.2000 | BRD-A36630025 | VCAP | 0.35μM | 6 h |
| 15 | 0.2000 | 70970 | PC3 | 10.0μM | 24 h |
| 16 | 0.2000 | BRD-K76674262 | SKB | 10.0μM | 24 h |
| 17 | 0.2000 | BRD-K06666320 | MCF7 | 10.0μM | 24 h |
| 18 | 0.2000 | trametinib | HT29 | 0.37μM | 24 h |
| 19 | 0.1333 | Homoharringtonine | HA1E | 10.0μM | 24 h |
| **Supplementary Table S3 (continued)** | | | | | |
| Rank | score | Perturbation | Cell line | Dose | Time |
| 20 | 0.1333 | cyclosporin A | HCC515 | 10.0μM | 24 h |
| 21 | 0.1333 | GR 32191 hydrochloride | HCC515 | 10.0μM | 24 h |
| 22 | 0.1333 | Rottlerin | HCC515 | 10.0μM | 24 h |
| 23 | 0.1333 | Syk Inhibitor | HCC515 | 10.0μM | 24 h |
| 24 | 0.1333 | AG-879 | HCC515 | 10.0μM | 24 h |
| 25 | 0.1333 | GSK-3b Inhibitor VIII | HCC515 | 10.0μM | 24 h |
| 26 | 0.1333 | ARP 101 | HCC515 | 10.0μM | 24 h |
| 27 | 0.1333 | SCH 58261 | HCC515 | 10.0μM | 24 h |
| 28 | 0.1333 | Piretanide | HCC515 | 10.0μM | 24 h |
| 29 | 0.1333 | cyclosporin A | PC3 | 10.0μM | 24 h |
| 30 | 0.1333 | IKK 16 | PC3 | 10.0μM | 24 h |
| 31 | 0.1333 | AG-879 | PC3 | 10.0μM | 24 h |
| 32 | 0.1333 | IMD 0354 | PC3 | 10.0μM | 24 h |
| 33 | 0.1333 | PI 828 | PC3 | 10.0μM | 24 h |
| 34 | 0.1333 | IKK 16 | PC3 | 10.0μM | 6 h |
| 35 | 0.1333 | IMD 0354 | VCAP | 10.0μM | 24 h |
| 36 | 0.1333 | BRD-K80348542 | HA1E | 10.0μM | 24 h |
| 37 | 0.1333 | PKC-412 | HCC515 | 10.0μM | 24 h |
| 38 | 0.1333 | geldanamycin | HA1E | 10.0μM | 24 h |
| 39 | 0.1333 | LOVASTATIN | HCC515 | 10.0μM | 24 h |
| **Supplementary Table S3 (continued)** | | | | | |
| Rank | score | Perturbation | Cell line | Dose | Time |
| 40 | 0.1333 | CGK 733 | PC3 | 10.0μM | 24 h |
| 41 | 0.1333 | CGP 71683 hydrochloride | PC3 | 10.0μM | 24 h |
| 42 | 0.1333 | EMETINE | HA1E | 10.0μM | 24 h |
| 43 | 0.1333 | TERFENADINE | HCC515 | 10.0μM | 24 h |
| 44 | 0.1333 | wortmannin | PC3 | 10.0μM | 24 h |
| 45 | 0.1333 | NICLOSAMIDE | VCAP | 10.0μM | 24 h |
| 46 | 0.1333 | LASALOCID SODIUM | HA1E | 10.0μM | 24 h |
| 47 | 0.1333 | RHODOMYRTOXIN B | HT29 | 10.0μM | 24 h |
| 48 | 0.1333 | RHODOMYRTOXIN B | PC3 | 10.0μM | 24 h |
| 49 | 0.1333 | Cimaterol | VCAP | 10.0μM | 6 h |
| 50 | 0.1333 | BRD-A18763547 | A375 | 10.0μM | 24 h |

Note: Three components derived from TCM (Rottlerin, CuI, and RHODOMYRTOXIN B) ranked among the top 50 candidates and were selected for subsequent studies.

Abbreviations: L1000CDS^2^, LINCS L1000 characteristic direction signatures search engine; TCM, traditional Chinese medicine; CuI, cucurbitacin I.

**Supplementary Table S4.** Molecular docking of three TCM-derived components with 20 PBs.

| Number | Component | Target protein (PBs) | Binding energy (kcal/mol) |
| --- | --- | --- | --- |
| 1 | CuI | RPS10 | -6.90 |
| 2 | CuI | LZTR1 | -8.80 |
| 3 | CuI | CMC2 | -5.95 |
| 4 | CuI | PSMA6 | -7.65 |
| 5 | CuI | CCZ1B | -6.25 |
| 6 | CuI | CNPY2 | -5.18 |
| 7 | CuI | IRF9 | -4.08 |
| 8 | CuI | EMC6 | -7.20 |
| 9 | CuI | LRCH4 | -5.44 |
| 10 | CuI | SDHA | -9.44 |
| 11 | CuI | CRLS1 | -7.92 |
| 12 | CuI | MRPL2 | -6.95 |
| 13 | CuI | TTLL3 | -6.24 |
| 14 | CuI | NDUFA9 | -7.53 |
| 15 | CuI | WDR27 | -6.41 |
| 16 | CuI | PDE4DIP | -3.80 |
| 17 | CuI | CSE1L | -6.23 |
| 18 | CuI | PERP | -6.24 |
| **Supplementary Table S4 (continued)** | | | |
| Number | Component | Target protein (PBs) | Binding energy (kcal/mol) |
| 19 | CuI | S100A11 | -6.39 |
| 20 | CuI | SLC25A6 | -5.58 |
| 21 | Rottlerin | RPS10 | -5.12 |
| 22 | Rottlerin | LZTR1 | -5.50 |
| 23 | Rottlerin | CMC2 | -5.43 |
| 24 | Rottlerin | PSMA6 | -5.29 |
| 25 | Rottlerin | CCZ1B | -4.41 |
| 26 | Rottlerin | CNPY2 | -2.91 |
| 27 | Rottlerin | IRF9 | -3.08 |
| 28 | Rottlerin | EMC6 | -5.83 |
| 29 | Rottlerin | LRCH4 | -4.84 |
| 30 | Rottlerin | SDHA | -6.93 |
| 31 | Rottlerin | CRLS1 | -6.70 |
| 32 | Rottlerin | MRPL2 | -4.98 |
| 33 | Rottlerin | TTLL3 | -4.59 |
| 34 | Rottlerin | NDUFA9 | -6.84 |
| 35 | Rottlerin | WDR27 | -4.61 |
| 36 | Rottlerin | PDE4DIP | -3.83 |
| 37 | Rottlerin | CSE1L | -4.13 |
| **Supplementary Table S4 (continued)** | | | |
| Number | Component | Target protein (PBs) | Binding energy (kcal/mol) |
| 38 | Rottlerin | PERP | -5.17 |
| 39 | Rottlerin | S100A11 | -5.19 |
| 40 | Rottlerin | SLC25A6 | -4.31 |
| 41 | RHODOMYRTOXIN B | RPS10 | -5.43 |
| 42 | RHODOMYRTOXIN B | LZTR1 | -6.18 |
| 43 | RHODOMYRTOXIN B | CMC2 | -6.10 |
| 44 | RHODOMYRTOXIN B | PSMA6 | -7.05 |
| 45 | RHODOMYRTOXIN B | CCZ1B | -5.47 |
| 46 | RHODOMYRTOXIN B | CNPY2 | -3.56 |
| 47 | RHODOMYRTOXIN B | IRF9 | -3.12 |
| 48 | RHODOMYRTOXIN B | EMC6 | -5.33 |
| 49 | RHODOMYRTOXIN B | LRCH4 | -4.70 |
| 50 | RHODOMYRTOXIN B | SDHA | -6.59 |
| 51 | RHODOMYRTOXIN B | CRLS1 | -6.33 |
| 52 | RHODOMYRTOXIN B | MRPL2 | -5.37 |
| 53 | RHODOMYRTOXIN B | TTLL3 | -5.00 |
| 54 | RHODOMYRTOXIN B | NDUFA9 | -7.32 |
| 55 | RHODOMYRTOXIN B | WDR27 | -5.63 |
| 56 | RHODOMYRTOXIN B | PDE4DIP | -4.15 |
| **Supplementary Table S4 (continued)** | | | |
| Number | Component | Target protein (PBs) | Binding energy (kcal/mol) |
| 57 | RHODOMYRTOXIN B | CSE1L | -5.56 |
| 58 | RHODOMYRTOXIN B | PERP | -4.83 |
| 59 | RHODOMYRTOXIN B | S100A11 | -4.86 |
| 60 | RHODOMYRTOXIN B | SLC25A6 | -6.20 |

Note: Binding energy cutoffs: ≤ -7 kcal/mol (strong binding), -7 to -4 kcal/mol (moderate binding), > -4 kcal/mol (weak binding).

**Supplementary Table S5.** Display of the top 5 PB-CuI docking complexes ranked by binding energy.

| Component | Rank | SPB target | Binding Energy (kcal/mol) |
| --- | --- | --- | --- |
|  | 1 | SDHA | -9.44 |
| CuI | 2 | LZTR1 | -8.80 |
|  | 3 | CRLS1 | -7.92 |
|  | 4 | PSMA6 | -7.65 |
|  | 5 | NDUFA9 | -7.53 |

Note: CuI exhibited the strongest binding energy among the three candidate components and was selected for subsequent study.
